# Kindlin-2 modulates MafA and β-catenin expression to regulate β-cell function and mass in mice

**DOI:** 10.1038/s41467-019-14186-y

**Published:** 2020-01-24

**Authors:** Ke Zhu, Yumei Lai, Huiling Cao, Xiaochun Bai, Chuanju Liu, Qinnan Yan, Liting Ma, Di Chen, Giedrius Kanaporis, Junqi Wang, Luyuan Li, Tao Cheng, Yong Wang, Chuanyue Wu, Guozhi Xiao

**Affiliations:** 1 Department of Orthopedic Surgery, Rush University Medical Center, Chicago, IL 60612 USA; 2 Guangdong Provincial Key Laboratory of Cell Microenvironment and Disease Research, Shenzhen Key Laboratory of Cell Microenvironment, and Department of Biology, Southern University of Science and Technology, 518055 Shenzhen, China; 3 Department of Cell Biology, School of Basic Medical Sciences, Southern Medical University, 510515 Guangzhou, China; 4 Department of Orthopedic Surgery, New York University School of Medicine, New York, NY 10003 USA; 5 Department of Cell Biology, New York University School of Medicine, New York, NY 10016 USA; 6 Department of Molecular Biophysics and Physiology, Rush University Medical Center, Chicago, IL 60612 USA; 7 State Key Laboratory of Medicinal Chemical Biology and Nankai University College of Pharmacy, 300071 Tianjin, China; 8 State Key Laboratory of Experimental Hematology, Institute of Hematology and Blood Disease Hospital, Center for Stem Cell Medicine, Chinese Academy of Medical Sciences & Peking Union Medical College, 300020 Tianjin, China; 9 UVA Islet Microfluidic Laboratory, Department of Surgery, the University of Virginia, Charlottesville, VA 22908 USA; 10 Department of Pathology, University of Pittsburgh, Pittsburgh, PA 15261 USA; 11 Department of Orthopedic Surgery, Rush University Medical Center, Chicago, IL 60612 USA

**Keywords:** Biochemistry, Cell biology

## Abstract

β-Cell dysfunction and reduction in β-cell mass are hallmark events of diabetes mellitus. Here we show that β-cells express abundant Kindlin-2 and deleting its expression causes severe diabetes-like phenotypes without markedly causing peripheral insulin resistance. Kindlin-2, through its C-terminal region, binds to and stabilizes MafA, which activates insulin expression. Kindlin-2 loss impairs insulin secretion in primary human and mouse islets in vitro and in mice by reducing, at least in part, Ca^2+^ release in β-cells. Kindlin-2 loss activates GSK-3β and downregulates β-catenin, leading to reduced β-cell proliferation and mass. Kindlin-2 loss reduces the percentage of β-cells and concomitantly increases that of α-cells during early pancreatic development. Genetic activation of β-catenin in β-cells restores the diabetes-like phenotypes induced by Kindlin-2 loss. Finally, the inducible deletion of β-cell Kindlin-2 causes diabetic phenotypes in adult mice. Collectively, our results establish an important function of Kindlin-2 and provide a potential therapeutic target for diabetes.

## Abstract (web summary)

Beta cell dysfunction and reduction in beta cell mass are hallmark events in the pathogenesis of diabetes mellitus. We identify focal adhesion protein Kindlin-2 as a key factor that controls insulin synthesis and secretion and beta cell mass by modulating MafA and beta-catenin proteins in pancreatic beta cells.

## Introduction

Diabetes mellitus (DM) is a group of metabolic diseases characterized by prolonged hyperglycemia with complications involving multiple critical organs. Both the incidence and prevalence of DM are high and steadily increasing worldwide. DM results from dysfunction and death of the insulin-producing pancreatic β-cells. Understanding the mechanisms that regulate processes, such as insulin expression and secretion as well as maintenance of β-cell mass, are of paramount biological and clinical importance, as they explain how these processes regulate metabolic homeostasis under physiological conditions and how their activities malfunction under diseased states, such as DM.

The transcription of the insulin gene is regulated by a number of factors, including MafA, Pax6, Pdx1, and Beta2^1–4^. Among those, MafA, a member of the Maf family of basic-leucine zipper transcription factors that is exclusively expressed in the pancreatic β-cells and len^5–7^, is a key regulator in coordinating and controlling insulin gene expression^8^. Interestingly, MafA is also required for the expression of Pdx1 and Beta2^9^. Furthermore, MafA interacts with Pdx1 and Beta2 and synergistically activates insulin gene transcription^8^. In mice, loss of MafA causes glucose intolerance, leading to development of DM^9^. While the role of MafA in regulation of insulin expression is well documented in the literature, how its expression and activity are modulated is incompletely understood.

The secretion of insulin from β-cells is exquisitely regulated to meet metabolic demand through complex mechanisms that involve the integration and interaction of multiple external and internal stimuli. Blood glucose is the primary stimulus of insulin secretion from β-cells. After being taken up by β-cells through glucose transporter 2, it stimulates insulin release through exocytosis by a process involving the influx of calcium^10^. While insulin resistance, a condition in which peripheral muscle and adipose tissues fail to respond to insulin properly, is necessary to cause type 2 diabetes (T2D), a decline in β-cell function, as demonstrated by inadequate insulin secretion in response to elevated blood glucose levels, becomes a hallmark of disease progression^11,12^. Furthermore, inadequate β-cell mass leads to insulin insufficiency, a contributor to the onset of hyperglycemia in both type 1 diabetes and T2D. Rulifson et al. reported that β-catenin expression is critical for β-cell proliferation and expansion and the maintenance of β-cell mass^13^. Key signals that control insulin secretion are poorly understood.

During pancreatic development and homeostasis, β-cell precursors must migrate to proper locations to form the islets of Langerhans and establish a suitable β-cell mass. Furthermore, β-cells must communicate with their extracellular matrix (ECM) to express and secret insulin in order to maintain glucose homeostasis. Kindlins are evolutionarily conserved cytoplasmic proteins that are key regulators of integrin-mediated cell-ECM adhesion, migration, and signaling^14–19^. In mammals, the Kindlin family consists of Kindlin-1, -2, and -3. They are encoded by three different genes, Kindlin-1 by *Fermt1*, Kindlin-2 by *Fermt2*, and Kindlin-3 by *Fermt3*^14,15,20,21^. Each Kindlin protein contains a FERM (F for 4.1 protein, E for ezrin, R for radixin, and M for moesin) domain that is responsible for interacting with β-integrin cytoplasmic tails^14,16,22–27^. Bledzka et al. reported that Kindlin-2 binds actin and regulates integrin outside-in signaling^28^. Genetic studies in humans reveal that mutations in the *FERMT1* gene lead to Kindler syndrome, which is characterized by skin blistering^21,29^. Mutations in the *FERMT3* gene impair integrin activation in humans, resulting in leukocyte adhesion deficiency-III, severe bleeding, frequent infections, and osteopetrosis^30–33^. Global inactivation of *Fermt2* in mice results in early embryonic lethality at E7.5^22^. Conditional deletion of *Fermt2* selectively in head and limb mesenchymal progenitors in mice causes severe chondrodysplasia and complete loss of the skull vault by impairing TGF-β signaling and Sox9 expression^34^. Zhang et al. showed that postnatal loss of Kindlin-2 causes progressive heart failure^35^. Our recent study demonstrated that Kindlin-2 associates with Rho GDP-dissociation Inhibitor α to suppress Rac1 activation and regulate podocyte structure and function in mice^18^.

In this study, we use a conditional knockout strategy to delete Kindlin-2 expression in β-cells during pancreatic development in mice. Results from comprehensive analyses of control and mutant mice demonstrate a critical role for Kindlin-2 in regulation of β-cell function and mass. In vitro and in vivo studies reveal that Kindlin-2 loss dramatically reduces insulin expression and secretion and impairs β-cell proliferation and mass, resulting in severe diabetes-like phenotypes. Kindlin-2 ablation markedly alters the islet composition by decreasing the percentage of β-cells and concomitantly increasing that of α-cells during embryonic development. Mechanistically, Kindlin-2 activates insulin gene expression by interacting with and stabilizing MafA protein. Furthermore, Kindlin-2 loss activates GSK-3β and downregulates β-catenin. Inducible deletion of Kindlin-2 in β-cells in adult mice causes similar diabetic phenotypes with impaired glucose tolerance and glucose-stimulated insulin secretion (GSIS), which are largely reversed by genetic upregulation of β-catenin in β-cells. Thus, we demonstrate that Kindlin-2, through its expression in β-cells, regulates glucose homeostasis by modulating insulin expression and secretion and β-cell mass through distinct molecular mechanisms.

## Results

### Kindlin-2 is highly expressed in pancreatic β-cells

**Fig. 1:**
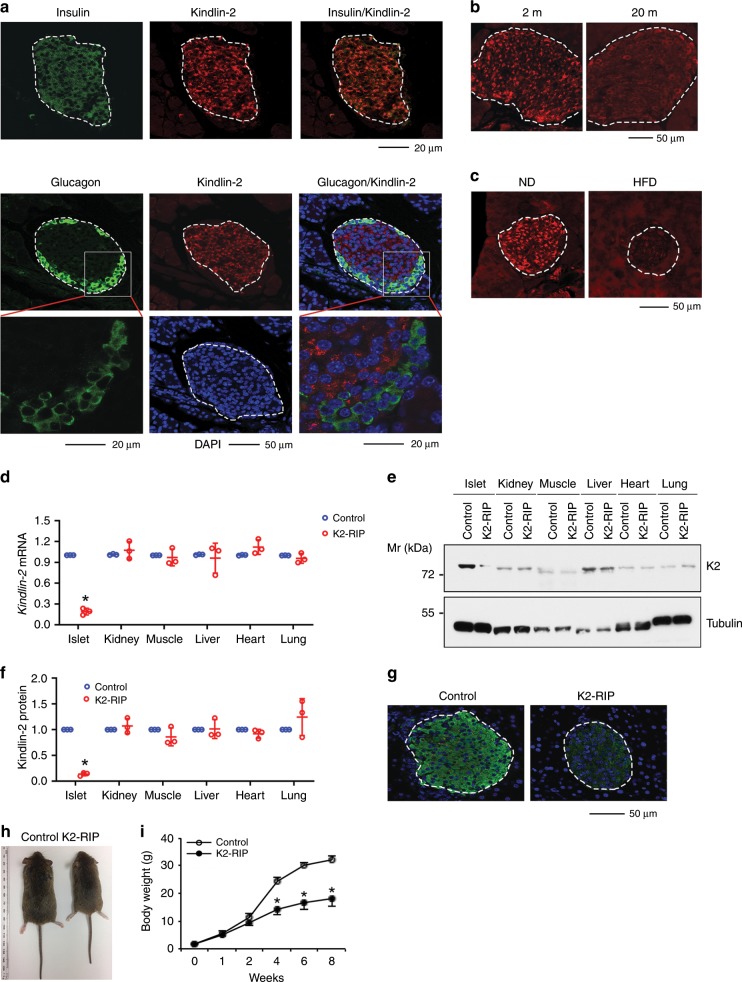
Kindlin-2 is highly expressed in β-cells and Kindlin-2 loss results in a growth retardation in mice. **a** Immunofluorescent (IF) staining. Sections of 2-month-old mouse pancreas were stained with anti-Kindlin-2 antibody, anti-insulin antibody, or anti-glucagon antibody (Sigma, G2654). Scale bar, 20 or 50 μm as indicated. **b** IF staining of 2- (left) and 20-month-old (right) mouse pancreatic sections with Kindlin-2 antibody. Scale bar, 50 μm. **c** IF of pancreatic sections from mice treated with normal diet (ND) or high-fat diet (HFD) with Kindlin-2 antibody. Scale bar, 50 μm. **d** Quantitative real-time reverse transcriptase-polymerase chain reaction (qPCR) analyses. Total RNAs isolated from the indicated tissues of 2-month-old male *K2-RIP* mice or control littermates (*RIP-Cre*) were subjected to qPCR analyses for Kindlin-2 expression. *Kindlin-2* mRNA was normalized to *Gapdh* mRNA. Statistical analyses (Student’s *t* test) were performed using the average values of triplicates from three independent experiments. *P < 0.05, versus control. **e**, **f** Western blot analyses. Protein extracts isolated from the indicated tissues of 2-month-old male *K2-RIP* mice or control littermates (*RIP-Cre*) were subjected to Western blot analyses for Kindlin-2 expression. α-Tubulin was used as a loading control. Quantitative data (**f**). Statistical analyses (Student’s *t* test) were performed using the average values of triplicates from three independent experiments. *P < 0.05, versus control. **g** IF staining of pancreatic sections of 2-month-old male *K2-RIP* mice or control littermates (*RIP-Cre*) with Kindlin-2 antibody. Scale bar, 50 μm. **h** Gross appearance of 2-month-old male *K2-RIP* mice and control littermates (*RIP-Cre*). **i** Animal growth curve. *N* *=* 12 for control, *N* *=* 11 mice *K2-RIP*, *P < 0.05, versus control, Student’s *t* test. Results are expressed as mean ± standard deviation. Source data for **d**–**f** are provided as a Source Data file.

To investigate the potential role of Kindlin-2 in the pancreas, we performed immunofluorescent (IF) staining of mouse pancreatic sections using specific antibodies against Kindlin-2, glucagon, and insulin and observed that Kindlin-2 protein was highly expressed in the insulin-expressing β-cells, but not in the glucagon-expressing α-cells located in the outer rim of the pancreatic islets (Fig. 1a). Furthermore, Kindlin-2 was weakly expressed in cells outside the islets (Fig. 1a). Kindlin-2 expression was markedly reduced in islets from aging (20-month-old) or high-fat diet-treated mice (Fig. 1b, c).

### Kindlin-2 loss causes severe diabetes-like phenotypes

The β-cell-specific expression of Kindlin-2 observed above prompted us to investigate whether Kindlin-2 plays a role in β-cells. To do this, we deleted Kindlin-2 expression in β-cells by breeding the floxed Kindlin-2 (*Kindlin-2*^*fl/fl*^) mice, in which exons 5 and 6 of the *Kindlin-2* gene are flanked by two loxP sites^34^, with the *RIP-Cre* transgenic mice, in which the 668-bp rat insulin II gene promoter (*RIP*) drives Cre expression in β-cells^36^, resulting in the β-cell conditional knockout mice, i.e., the *Kindlin-2*^*fl/fl*^*; RIP-Cre* mice (hereafter referred to as *Kindlin-2*^*RIP*^ or *K2-RIP*). We performed the quantitative real-time reverse transcriptase-polymerase chain reaction (qPCR) analysis using RNAs isolated from islets of the two genotypes and observed that the level of *Kindlin-2* mRNA was dramatically reduced in islets of *K2-RIP* mice relative to control littermates (Fig. 1d). Results from Western blotting (Fig. 1e, f) and IF staining of pancreatic sections (Fig. 1g) revealed that the level of Kindlin-2 protein was drastically decreased in *K2-RIP* relative to control littermate islets. This reduction is specific because the Kindlin-2 expression in nonpancreatic tissues, such as kidney, muscle, liver, heart, and lung, was not significantly different in *K2-RIP* mice relative to control littermates (Fig. 1d–f). *K2-RIP* mice were born at the expected Mendelian frequency, and, starting at 2 weeks of age, displayed growth retardation (Fig. 1h, i).

**Fig. 2:**
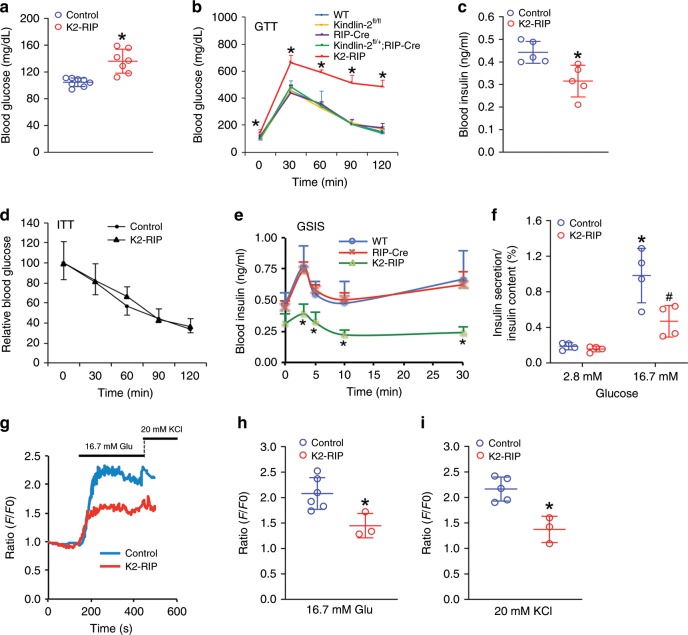
Kindlin-2 loss causes severe diabetes-like phenotypes without affecting insulin sensitivity. **a** Fasting blood glucose level. Two-month-old male control (*RIP-Cre*) and *K2-RIP* mice were fasted overnight. *P < 0.05, versus control, *N* *=* 8 for control, *N* *=* 7 *K2-RIP*, **b** GTT. Mice treated as in **a** were given intraperitoneal injections of glucose (2 g/kg body weight). *P < 0.05, versus control, *N* *=* 5 for WT and *K2-RIP*, *N* *=* 6 for *Kindlin-2*^*fl/fl*^, and *N* *=* 7 for *RIP-Cre* and *Kindlin-2*^*fl/+*^*; RIP-Cre*. **c** Blood insulin level. Mice were treated as in **a**. *P < 0.05, versus control, *N* = 5 mice per genotype. **d** ITT. Mice fasted (6–7 h) were intraperitoneally injected with a single dose of recombinant human insulin (1 U/kg body weight). *P < 0.05, versus control, *N* = 6 for control, *N* = 8 for *K2-RIP*. **e** In vivo GSIS. Mice treated as in **a** and intraperitoneally injected with glucose (2 g/kg body weight). Blood insulin levels were measured by ELISA at 0, 3, 5, 10, and 30 min after glucose injection. *P < 0.05, versus control, *N* *=* 5 for WT, *N* *=* 8 for *RIP-Cre* and *K2-RIP*. **f** In vitro GSIS. Islets isolated from 2-month-old male control *K2-RIP* mice were treated with 2.8 or 16.7 mM glucose. Amounts of insulin in supernatant were measured by ELISA assay. Proteins were extracted and total insulin content was measured. Supernatant insulin was normalized to total insulin content. *P < 0.05, versus 2.8 mM glucose, ^#^*P* < 0.05, versus control. **g** Ca^2+^ influx. Representative results from control and *K2-RIP* islets are provided. *N* = 15 control islets, *N* = 20 *K2-RIP* islets. **h** Quantification of glucose (16.7 mM) stimulation in control and mutant islets. *N* = 6 islets for control, *N* = 3 islets for *K2-RIP*, *P < 0.05, versus control. **i** Quantification of KCI (20 mM) stimulation in control and mutant islets. *N* = 5 islets for control, *N* = 3 islets for *K2-RIP*, *P < 0.05, versus control. Results are expressed as mean ± standard deviation (s.d.) and Student’s *t* test was used in this figure. Source data for **a**–**f**, **h**, **i** are provided as a Source Data file.

We next measured the level of fasting blood glucose and observed a significant elevation in 2- and 4-month-old male *K2-RIP* mice compared with age- and sex-matched control littermates (*P* < 0.05, Student’s *t* test) (Fig. 2a and Supplementary Fig. 1a). We further performed the glucose tolerance test (GTT). Mice were given a single intraperitoneal (i.p.) injection of glucose (2 g/kg body weight) and blood samples were then taken to determine how quickly glucose was cleared from the blood. Results revealed that the blood glucose levels were significantly increased in 2- and 4-month-old male *K2-RIP* mice compared with sex- and age-matched control groups at all time points (Fig. 2b and Supplementary Fig. 1b). The littermates generated from breeding, including the wild-type (WT) mice that contain no Cre or floxed Kindlin-2 genes, Kindlin-2 flox heterozygotes that harbor *RIP-Cre* (i.e., *RIP-Cre; Kindlin-2*^*fl/+*^), Cre-negative floxed Kindlin-2 mice (i.e., *Kindlin-2*^*fl/fl*^) or Cre transgenic mice (i.e., *RIP-Cre*), were viable and fertile and did not display marked glucose intolerance (Fig. 1b). These mice were used as control groups as specified in each experiment. A similar glucose intolerance was observed in 2-month-old female *K2-RIP* mice (Supplementary Fig. 1c). Kindlin-2 loss significantly reduced the level of fasting blood insulin (*P* *<* 0.05, *K2-RIP* versus control, Student’s *t* test) (Fig. 2c and Supplementary Fig. 1d, e). We further performed insulin tolerance tests (ITT) and observed no significant difference in insulin sensitivity between the two genotypes (Fig. 2d and Supplementary Fig. 1f).

We next performed the GSIS assays to investigate whether Kindlin-2 regulates insulin secretion in mice. The blood insulin levels in *K2-RIP* mice and control littermates were measured at 3, 5, 10, and 30 min after a single-glucose injection (2 g/kg body weight). Both WT and *RIP-Cre* mice displayed an initial burst of insulin secretion within 5 min of glucose injection (the first-phase response), followed by a more sustained insulin secretion (the second-phase response). Both the first- and second-phase responses were markedly impaired in *K2-RIP* mice compared with WT and *RIP-Cre* littermates (Fig. 2e). To further establish the requirement for Kindlin-2 in insulin secretion, primary islets isolated from *K2-RIP* mice and control littermates (*RIP-Cre*) were treated with 2.8 or 16.7 mM glucose in vitro, followed by measurements of insulin protein in the supernatant and in the whole cell extracts. Treatment of 16.7 mM glucose stimulated a 5.2-fold insulin secretion in control islets; this value was decreased to threefold in *K2-RIP* islets (Fig. 2f). Kindlin-2 loss impaired both the glucose- and KCl-induced Ca^2+^ release from islets. After stimulation with a 16.7 mM glucose load, Ca^2+^ release was significantly reduced in *K2-RIP* versus control islets (Fig. 2g, h). Similarly, the amplitude of Ca^2+^ transients stimulated by a 20 mM KCl load was also significantly decreased in *K2-RIP* islets compared with that of control islets (Fig. 2g, i).

### Kindlin-2 loss reduces β-cell proliferation and mass

**Fig. 3:**
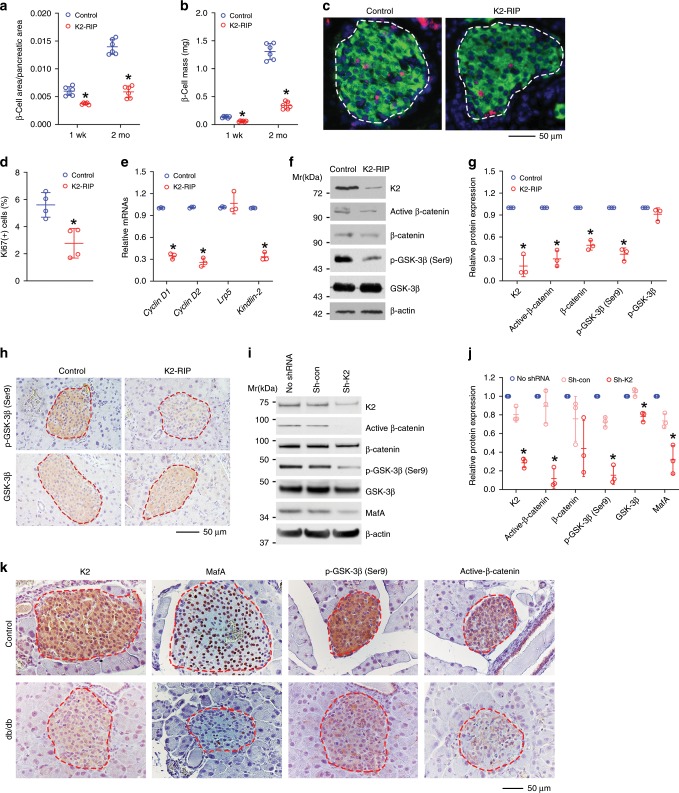
Kindlin-2 loss activates GSK-3β and reduces β-catenin expression and β-cell proliferation and mass. **a**, **b** Pancreatic sections from 1-week- or 2-month-old male control (*RIP-Cre*) and *K2-RIP* mice were subjected to IF staining for insulin, followed by measurements of β-cell area/pancreatic area ratio (**a**) and β-cell mass (**b**). *P < 0.05, versus control, *N* *=* 5 for 2-month-old *K2-RIP* in **a**, *N* *=* 6 for the remaining groups, Student’s *t* test. **c**, **d** Cell proliferation. Sections of 1-week-old control and mutant male pancreas were double stained with antibodies against insulin and Ki67 (**c**). Ki67-positive cells in islets were normalized to total insulin-positive cells in the same area (**d**). Around 444–816 β-cells (insulin-positive cells) per mouse were counted. *N* *=* 4 mice per genotype. Scale bar, 20 μm. *P < 0.05, versus control, Student’s *t* test. **e** qPCR analyses. Total RNAs isolated from islets of 1-week-old *K2-RIP* and control littermates were subjected to qPCR analysis for the indicated genes. mRNA levels were normalized to *Gapdh* mRNA. Statistical analyses (Student’s *t* test) were performed using the average values of triplicates from three independent experiments. *P < 0.05, versus control. **f**, **g** Western blot analysis. Protein extracts isolated from islets isolated from *K2-RIP* mice and control littermates (*RIP-Cre*) were subjected to Western blot analyses with indicated antibodies. Quantitative data from three independent experiments (**g**). *P < 0.05, versus control, Student’s *t* test. **h** IHC staining of pancreatic sections with antibodies against phospho-GKS-3β(Ser9) (top) and total GSK-3β (bottom). Scale bar, 50 μm. **i**, **j** Western blotting. Human pancreatic islets were infected with or without Kindlin-2 shRNA (Sh-K2) or control (Sh-con) lentiviruses, followed by Western blot analyses with indicated antibodies. Quantitative data from three independent experiments (**j**). *P < 0.05, versus control (Sh-con), Student’s *t* test. **k** IHC staining of pancreatic sections of 3-month-old *db/db* mice and age- and sex-matched C57BL/6 (control) mice with indicated antibodies. *N* = 3 mice per genotypes. Scale bar, 50 μm. Results are expressed as mean ± standard deviation. Source data for **a**, **b**, **d**–**f**, **i**, **j** are provided as a Source Data file.

We further measured the β-cell area/pancreatic area ratio and β-cell mass of *K2-RIP* and control littermates and demonstrated that both parameters were dramatically reduced in 1-week-old and 2-month-old *K2-RIP* mice compared with those of their age- and sex-matched control littermates (*Cre-RIP*) (Fig. 3a, b). Notably, the magnitude of reductions in both parameters was larger in 2-month-old mice than in 1-week-old *K2-RIP* mice (Fig. 3a, b), suggesting that impairment of β-cells becomes worse over time. Immunofluorescence (IF) staining of pancreatic sections using antibodies against Kindlin-2 and the proliferating cell nuclear antigen (Ki67) revealed that β-cell proliferation was reduced in 1-week-old *K2-RIP* mice relative to that in control littermates (Fig. 3c, d). The reduction in β-cell mass could contribute to the diabetic phenotypes in *K2-RIP* mice.

### Kindlin-2 loss activates GSK-3β and downregulates β-catenin

We further examined expression of cyclins D1 and D2, both required factors for β-cell proliferation^13^, by qPCR analysis and observed a reduced expression of both factors in 1-week-old *K2-RIP* relative to control islets (Fig. 3e). In contrast, expression of the Wnt co-receptor Lrp5 was not altered in *K2-RIP* islets compared with control islets (Fig. 3e). We further examined expression of β-catenin, which regulates cyclin D expression and β-cell proliferation and β-cell mass^13^ and observed that the levels of total and active β-catenin proteins were markedly reduced in *K2-RIP* islets compared with those of control islets (Fig. 3g). GSK-3 phosphorylates β-catenin and targets its degradation. It is known that phosphorylation of the Ser9 residue in the GSK-3β molecule decreases its kinase activity^37^. Interestingly, the level of phospho-GSK-3β (Ser9), but not total GSK-3β, was significantly reduced in *K2-RIP* relative to that of control islets (Fig. 3f, g). This finding was confirmed by in situ immunohistochemistry (IHC) staining of pancreatic sections of the two genotypes (Fig. 3h). We further knocked down Kindlin-2 expression in primary human pancreatic islets using a lentiviral Kindlin-2 shRNA and found that Kindlin-2 knockdown dramatically reduced the levels of MafA, p-GSK-3β (Ser9), and active β-catenin proteins (Fig. 3i, j). We also performed IHC staining of the pancreatic sections of 3-month-old diabetic *db/db* male mice and sex- and age-matched control mice and observed that the immunoreactivity of Kindlin-2, MafA, active β-catenin, and p-GSK-3β catenin was markedly reduced in *db/db* islets relative to that of control islets (Fig. 3k). Note: the 3-month-old *db/db* male mice displayed a severe diabetic phenotype, as demonstrated by a dramatic increase in the level of fasting blood glucose and abnormal GTT result (Supplementary Fig. 2).

### Kindlin-2 loss impairs pancreatic islet development

**Fig. 4:**
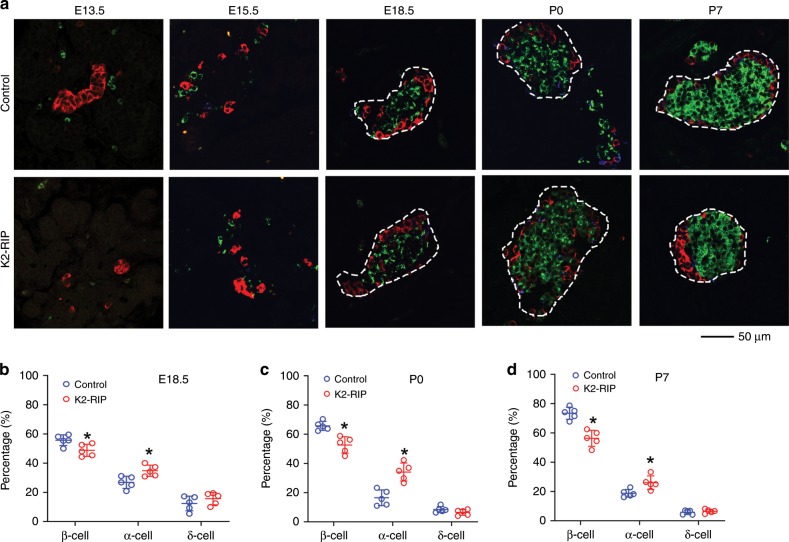
Kindlin-2 loss impairs pancreatic islet development. **a**–**d** Immunofluorescence (IF) staining. E13.5, E15.5, E18.5, P0, and P7 pancreatic sections of control and *K2-RIP* mice were subjected to IF staining using antibodies against insulin (for β-cell), glucagon (for α-cell), somatostatin (for δ-cell), or DAPI (not shown). Pictures were captured (**a**) and the total number of α-cells (red), β-cells (green), and δ-cells (blue) counted and expressed as percentages of total cells in islets (**b**–**d**). We measured the islet cell composition from 22 to 34 islets per mouse. We analyzed islets from two sides (each slide had 3–5 pancreatic sections on it) from each mouse and obtained an average value from each slide. Thus, we obtained two data points for each mouse. For statistical analysis, we used the average value of those two data points for each mouse. *N* = 5 mice per genotype at all time points. Scale bar, 50 μm. *P < 0.05, versus control, Student’s *t* test. Because islets were not completely formed in E13.5 and E15.5 mouse pancreatic tissue, we were unable to accurately define the borders of the islets and did not determine the islet composition at those two time points. Results are expressed as mean ± standard deviation. Source data for **b**–**d** are provided as a Source Data file.

We further determined the islet composition by performing IF staining of pancreatic sections of E13.5, E15.5, E18.5, P0, and P7 control and *K2-RIP* mice using antibodies against insulin (for β-cells), glucagon (for α-cells), or somatostatin (for δ-cells). The results showed, as expected, that the murine islets were composed primarily of β-cells clustered in a central core, surrounded by smaller numbers of α-cells and δ-cells in the periphery (Fig. 4a). The β-cells represented 55.8%, 65.7%, and 73.4% in E18.5, P0, and P7 control islets, which were reduced to 48.8%, 52.7%, and 56.4% in *K2-RIP* islets, respectively (Fig. 4b–d) (*P* < 0.05, control versus *K2-RIP* for all three time points). The percentages of α-cells represented 26.9%, 16.5%, and 18.7% in E18.5, P0, and P7 control islets, which were increased to 34.8%, 34.2%, and 26.1% in *K2-RIP* islets, respectively (Fig. 4b–d) (*P* < 0.05, control versus *K2-RIP* for all three time points). The percentages of δ-cells in islets were much smaller than those of α- or β-cells and were not significantly different between the two genotypes (Fig. 4b–d). Because islets were not completely formed in E13.5 and E15.5 mouse pancreatic tissue (Fig. 4a), we were unable to accurately define the borders of islets and did not determine the islet composition at those two time points.

### Kindlin-2 activates insulin gene expression

**Fig. 5:**
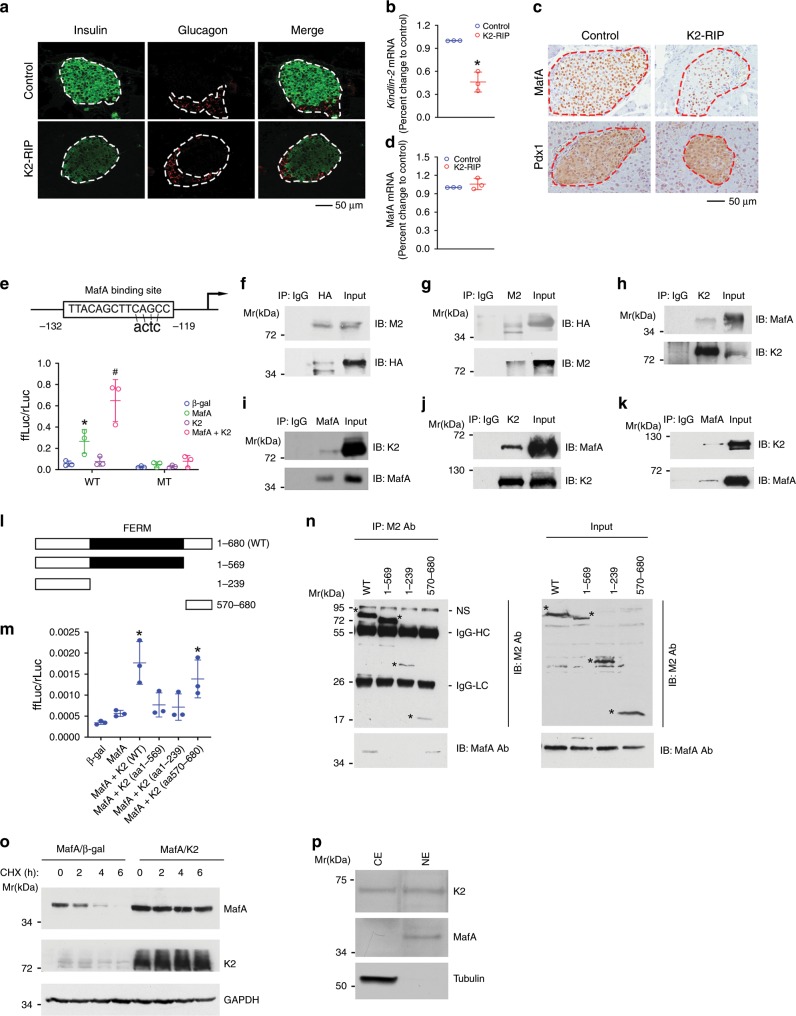
Kindlin-2 interacts with and stabilizes MafA to activate insulin gene expression. **a** IF staining. Pancreatic sections of 2-month-old male control (*RIP-Cre*) and *K2-RIP* mice were stained with the indicated antibodies. Scale bar, 50 μm. **b** qPCR analysis. Total RNA isolated from 2-month-old control and *K2-RIP* islets was subjected to qPCR analysis. *Insulin* mRNA was normalized to *Gapdh* mRNA. *P < 0.05, versus control. **c** IHC. Sections of 2-month-old pancreas were stained with the indicated antibodies. Scale bar, 50 μm. *N* = 5 per genotype. **d** qPCR analysis. Total RNA from **b** was used. **e** (top) Schematic representation of the rat *Ins1* gene promoter. **e** (bottom) COS-7 cells were transfected with p460rIns1-luc or p460rIns1C1mt-luc, pRL-SV40, and expression plasmids for β-gal, Kindlin-2, MafA, or Kindlin-2 plus MafA. After 48 h, cells were harvested for dual-luciferase assays. *P < 0.05, versus β-gal, ^#^*P* < 0.05, versus MafA. **f**–**k** IP assay. Whole cell extracts from COS-7 cells overexpressing pCMV/Flag-Kindlin-2 and pCMV/HA-MafA (**f**, **g**) or INS-1 cells (**h**, **i**) or a mixture of GST-MafA and GST-Kindlin-2 (**j**, **k**) were immunoprecipitated with the indicated antibodies, followed by Western blot analyses using the indicated antibodies. **l** A schematic showing the domain structure of Kindlin-2 and the deletion mutants. **m** COS-7 cells were co-transfected with p460rIns1-luc, pRL-SV40 (for normalization), pCMV/MafA, and pCMV vectors expressing wild type (WT) or various deletion Kindlin-2 mutants, followed by dual-luciferase assays. *P < 0.05, versus pCMV/β-gal. **n** IP assay. COS-7 cells were transfected with MafA expression plasmids and various Flag-Kindlin-2 deletion mutant expression plasmids. After 48 h, whole cell extracts were prepared and immunoprecipitated with M2 antibody, followed by Western blot analysis using M2 (top) or MafA antibody (bottom) (left panel). **o** COS-7 cells were transfected with a MafA expression plasmid with or without Kindlin-2 overexpression and treated with 10 µg/ml cycloheximide, followed by Western blotting at the indicated times. **p** Western blot analysis. Cytoplasmic (CE) and nuclear extracts (NE) from INS-1 cells subjected to Western blotting for Kindlin-2, MafA, and tubulin (a cytoplasmic protein). Results are expressed as mean ± standard deviation and Student’s *t* test was used in this figure. Source data for **b**, **d**–**k**, **m**–**p** are provided as a Source Data file.

We further investigated the potential role of Kindlin-2 in regulation of insulin expression. When compared with control islets, *K2-RIP* islets displayed reduced insulin, but not glucagon, expression, as demonstrated by IF (Fig. 5a) and qPCR analyses (Fig. 5b). In contrast, overexpression of Kindlin-2 increased expression of insulin protein and mRNA and activated the *Ins1* promoter in INS-1 cells, a rat pancreatic β-cell line (Supplementary Fig. 3). Because MafA is a master regulator of the insulin gene^9^, we determined the effect of Kindlin-2 deletion on its expression and found that MafA was strongly detected in the nuclei of control β-cells, which was dramatically reduced in *K2-RIP* β-cells (Fig. 5c, top). In contrast, expression of Pdx1, which regulates insulin gene transcription, was not decreased in *K2-RIP* β-cells (Fig. 5c, bottom). Because Kindlin-2 loss did not reduce the *MafA* mRNA level (Fig. 5d), this regulation must involve a posttranscriptional mechanism. The *Ins1* gene promoter contains a single MafA-binding site (C1) at −119/−132 (Fig. 5e, top) that is essential for its β-cell-specific expression^38,39^. As expected, the overexpression of MafA in INS-1 cells stimulated *Ins1* promoter activity and the introduction of a 4-bp point mutation into the C1 core sequence (from TACAGCTTCAGCC to TTACAGCTTCactc) abolished the MafA-dependent promoter activation (Fig. 5e, bottom). Kindlin-2 activation of the *Ins1* promoter was lost in the absence of MafA expression (Fig. 5e, bottom) and the C1 site mutation abolished the Kindlin-2 activation of the *Ins1* promoter (Fig. 5e, bottom).

### Kindlin-2 interacts with and stabilizes MafA protein

We next determined whether Kindlin-2 interacts with MafA by immunoprecipitation (IP) assays using extracts from COS-7 cells overexpressing Flag-Kindlin-2 and HA-MafA. Results showed that MafA protein was present in an anti-Kindlin-2 immunoprecipitate (Fig. 5f) and, vice versa, that Kindlin-2 protein was present in an anti-MafA immunoprecipitate (Fig. 5g). The interaction between the endogenous Kindlin-2 and MafA proteins was demonstrated by IP using extracts from rat pancreatic INS-1 cells (Fig. 5h, i). Results from the pull-down assays using purified GST-Kindlin-2 and glutathione S-transferase (GST)-MafA fusion proteins revealed a direct physical interaction between Kindlin-2 and MafA proteins (Fig. 5j, k). To identify domain(s) within the Kindlin-2 molecule necessary for interaction with MafA, we generated several deletion mutants of Kindlin-2 (Fig. 5l) and tested for their ability to activate *Ins1* promoter in the presence of MafA and observed a major drop in promoter activation when the C-terminus of Kindlin-2 (aa 570 to aa 680) was deleted (Fig. 5m). Further deletion to aa 239 did not exacerbate the activation of the *Ins1* promoter. The Kindlin-2 mutant with the N-terminal aa 1–569 deletion effectively activated the *Ins1* promoter. Consistent with this functional data, deletions of Kindlin-2 from aa 239 or 570 to aa 680 completely abolished the formation of the Kindlin-2-MafA complex (Fig. 5n). Likewise, deletion of aa 1 to aa 569 did not abrogate the interaction between Kindlin-2 and MafA. Together, these data establish that the aa 570–680 region of Kindlin-2 is required for its interaction with MafA. We next performed cycloheximide (a protein synthesis inhibitor) experiments and demonstrated that overexpression of Kindlin-2 markedly increased MafA stability (Fig. 5o). Western blot analysis using nuclear and cytoplasmic extracts showed that Kindlin-2 protein existed in the nuclei of INS-1 cells (Fig. 5p).

### Kindlin-2 functions independent of β integrin activation

Because the aa 570–680 region of Kindlin-2 contains a β integrin-binding site that mediates β integrin activation^40,41^, we next investigated whether Kindlin-2 regulation of MafA and insulin expression involves β integrin binding and activation. We determined the ability of wild-type Kindlin-2 (K2-WT) and an integrin-binding defective Kindlin-2 (K2-QW) to activate the *Ins1* promoter in the presence or absence of MafA in COS-7 cells. The results showed that K2-WT and K2-QW similarly activated *Ins1* promoter activity in a MafA-dependent manner (Supplementary Fig. 4a). Furthermore, both K2-WT and K2-QW could interact with and upregulate MafA (Supplementary Fig. 4b, c). Likewise, both K2-WT and K2-QW could activate endogenous *Ins1* mRNA expression in INS-1 cells (Supplementary Fig. 4d). Collectively, these results demonstrate that Kindlin-2 modulation of MafA and insulin expression is not dependent on its ability to activate β integrin.

### Kindlin-2 loss in adult mice causes diabetic phenotypes

**Fig. 6:**
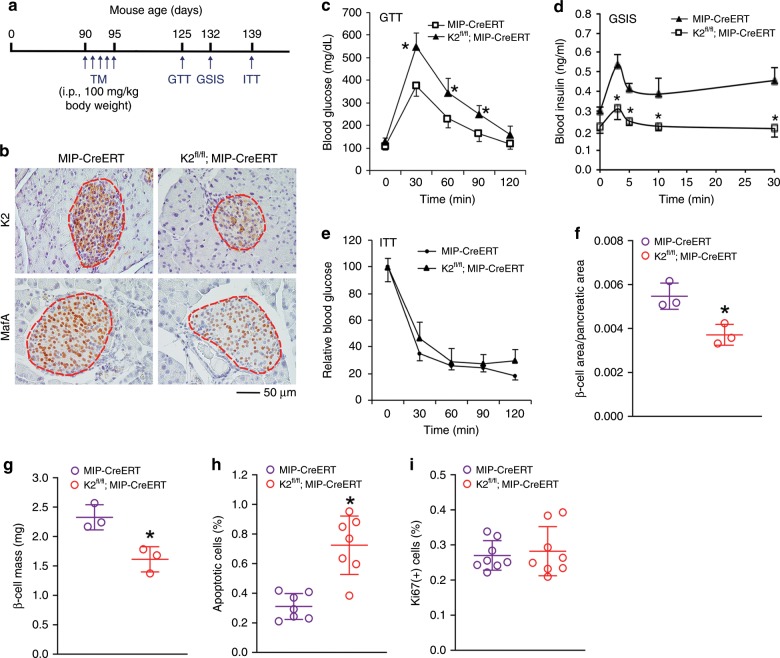
Inducible deletion of β-cell Kindlin-2 in adult mice leads to diabetes-like phenotypes. **a** Tamoxifen (TM) injection. **b** IHC. Three-month-old male *K2*^*f/f*^*; MIPCreERT* and control (MIP-CreERT) mice were treated with TM as described in **a**. After 30 days, pancreatic sections were stained with antibodies against Kindlin-2 or MafA. Scale bar, 50 μm. **c** GTT. Mice were treated with TM as described in **a**. Thirty days after the TM injection, mice were fasted overnight, and GTT assays were performed. *P < 0.05, versus control, *N* = 10 for control, *N* = 7 for *K2*^*f/f*^*; MIPCreERT*, Student’s *t* test. **d** Glucose stimulated insulin secretion (GSIS). Mice were treated with TM as in **a**. After 37 days, mice were fasted overnight, and GSIS assays were performed. *P < 0.05, versus control, *N* = 6 for both genotypes, Student’s *t* test. **e** Insulin tolerance test (ITT). Mice were treated with TM as in **a**. After 44 days, mice were fasted for 6–7 h, and ITT assays were performed. *N* = 5 for control, *N* = 4 for *K2*^*f/f*^*; MIPCreERT.* **f**, **g** β-Cell area/pancreatic area ratio and β-cell mass. Mice were treated as in **a**. After 30 days, pancreatic sections were subjected to IHC staining for insulin, followed by measurements of β-cell area/pancreatic area ratio (**f**) and β-cell mass (**g**). *P < 0.05, versus control, *N* = 3 per genotype. **h** Cell apoptosis. Mice were treated with TM as in **a**. After 30 days, pancreatic sections were subjected to insulin and TUNEL staining. Around 300–500 β-cells (insulin-positive cells) per mouse were counted. *N* = 7 mice per group. *P < 0.05, versus control, Student’s *t* test. **i** Cell proliferation. Mice were treated with TM as in **a**. After 30 days, pancreatic sections were double stained with antibodies against insulin and Ki67. Ki67-positive cells in islets were normalized to total insulin-positive cells in the same area (**i**). Around 300–500 β-cells per mouse were counted. *N* = 8 per genotype. Results are expressed as mean ± standard deviation. Source data for **c**–**i** are provided as a Source Data file.

We further investigated the role of Kindlin-2 in β-cells in adult animals. To do this, we utilized mice bearing conditional alleles of *Kindlin-2* (*Kindlin-2*^*fl/fl*^) and a *MIP-Cre/ERT* transgene, which expresses tamoxifen (TM)-inducible Cre recombinase under the control of mouse insulin gene 1 promoter (*MIP*) in β-cells. These mice (3-month-old) were treated with five daily injections of TM (100 mg/kg body weight) to delete Kindlin-2 expression in β-cells (hereafter referred to *as K2*^*f/f*^*; MIPCreERT*) (Fig. 6a). Consistent with results from *K2-RIP* mice, the expression of Kindlin-2 and MafA proteins was markedly reduced in islets of *as K2*^*f/f*^*; MIPCreERT* mice relative to that of control littermates, as demonstrated by IHC (Fig. 6b). At day 30 after TM injections, *K2*^*f/f*^*; MIPCreERT* mice displayed obvious abnormalities in GTT (Fig. 6c) and GSIS (Fig. 6d). A similar defect in GTT was observed in female *K2*^*f/f*^*; MIPCreERT* mice (Supplementary Fig. 5). *K2*^*f/f*^*; MIPCreERT* mice displayed a slightly steeper slope than control mice at 30 and 120 min, but not 60 and 90 min, after insulin injection, suggesting a slight reduction in insulin sensitivity in these animals (Fig. 6e). Furthermore, *K2*^*f/f*^*; MIPCreERT* mice displayed significant reductions in the β-cell area/pancreatic area ratio (Fig. 6f) and β-cell mass (Fig. 6g) and an increase in β-cell apoptosis (Fig. 6h). β-Cell proliferation was minimal and was not altered in adult *K2*^*f/f*^*; MIPCreERT* mice relative to that of control littermates (Fig. 6i).

### β-Catenin activation reverses the phenotypes of K2-RIP mice

**Fig. 7:**
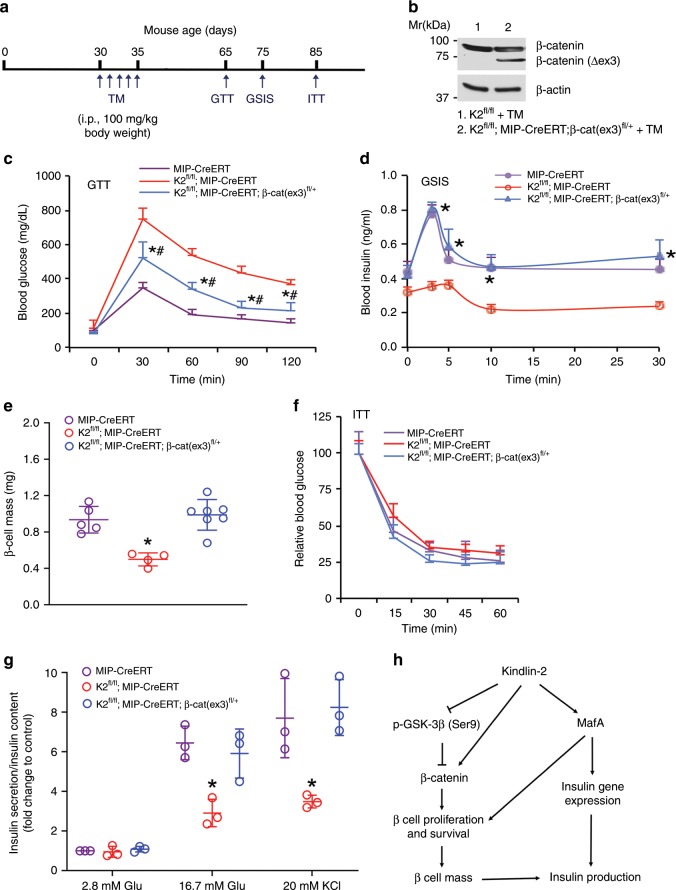
Genetic activation of β-cell β-catenin restores the diabetes-like phenotype induced by Kindlin-2 loss. **a** Tamoxifen (TM) injection. **b** Western blotting. Protein extracts from isolated islets of indicated genotypes treated with TM as described in **a** were subjected to Western blotting. **c** GTT. Mice were treated with TM as described in **a** and fasted overnight. *P < 0.05, versus *MIP-CreERT* (control), ^#^*P* < 0.05, versus *K2*^*f/f*^*; MIP-CreERT*, *N* = 6 for *MIP-CreERT*, *N* = 4 for *K2*^*f/f*^*; MIP-CreERT* and *K2*^*f/f*^*; MIP-CreERT;* *β-cat (ex)*^*fl/+*^, Student’s *t* test. **d** GSIS. Mice with indicated genotypes were treated with TM as described in **a** and fasted overnight. *P < 0.05, versus *K2*^*f/f*^*; MIP-CreERT*, *N* = 5 for *MIP-CreERT, N* = 4 for *K2*^*f/f*^*; MIP-CreERT*, and *N* = 6 for *K2*^*f/f*^*; MIP-CreERT;* *β-cat (ex)*^*fl/+*^. Student’s *t* test. **e** β-cell mass. After the ITT experiments, pancreatic sections were subjected to IHC staining for insulin, followed by measurements of β-cell mass. *P < 0.05, versus *MIP-CreERT* (control), ^#^*P* < 0.05, versus *K2*^*f/f*^*; MIP-CreERT*. *N* = 5 for *MIP-CreERT, N* = 4 for *K2*^*f/f*^*; MIP-CreERT, and N* = 7 for *K2*^*f/f*^*; MIP-CreERT;* *β-cat (ex)*^*fl/+*^. Student’s *t* test. **f** ITT. Mice with indicated genotypes were treated with TM as described in **a** and fasted (6–7 h), followed by performance of ITT assays. *N* = 7 for control, *N* = 4 for *K2*^*f/f*^*; MIP-CreERT* and *K2*^*f/f*^*; MIP-CreERT;* *β-cat (ex)*^*fl/+*^. **g** Insulin secretion. One-month-old male *MIP-CreERT*, *K2*^*f/f*^*; MIP-CreERT* and *K2*^*f/f*^*; MIP-CreERT;* *β-cat (ex)*^*fl/+*^ mice were injected with TM as described in **a**. One month after the last TM injection, islets were isolated from each group and treated with 2.8 or 16.7 mM glucose or 20 mM KCl. Amounts of insulin in supernatant were measured by enzyme-linked immunosorbent assay (ELISA). Insulin secretion was measured as described in Fig. 2f. *P < 0.05, versus control (*MIP-CreERT*), Student’s *t* test. Results are expressed as mean ± standard deviation. **h** A working model for Kindlin-2 regulation of β cell function. Source data for **b**–**g** are provided as a Source Data file.

We additionally investigated whether genetic activation of β-catenin in β-cells reverses the diabetic phenotypes induced by Kindlin-2 loss. To do this, we bred *K2*^*f/f*^*; MIPCreERT* with *β-catenin (ex3)*^*fl/fl*^ mice and generated *K2*^*f/f*^*; MIPCreERT;* *β-catenin (ex3)*^*fl/+*^ (hereafter referred to *as K2*^*f/f*^*; MIPCreERT;* *β-cat(ex3)*^*fl/+*^) mice. In these mice, a dominant active form of β-catenin that lacks the GSK phosphorylation sites (*β-catenin(ex3)*) was expressed in β-cells in the *K2*^*f/f*^*; MIPCreERT* genetic background. At 1 month of age, *K2*^*f/f*^*; MIPCreERT;* *β-cat(ex3)*^*fl/+*^ mice and control littermates were treated with TM as indicated in Fig. 7a. Western blotting detected the presence of the β-catenin(ex3) protein in islets of the *K2*^*f/f*^*; MIPCreERT;* *β-cat(ex3)*^*fl/+*^ mice treated with TM, but not in Cre-negative control mice treated with TM (Fig. 7b). The activation of β-catenin significantly restored the defective GTT in *K2*^*f/f*^*; MIPCreERT* mice (Fig. 7c) (*P* < 0.05, *K2*^*f/f*^*; MIP-CreERT* versus *K2*^*f/f*^*; MIP-CreERT; β-cat (ex3)*^*fl/+*^). Furthermore, the activation of β-catenin largely restored the abnormalities in GSIS and β-cell mass in *K2*^*f/f*^*; MIPCreERT* mice to levels comparable with those in the control group (Fig. 7d, e) without markedly affecting insulin sensitivity (Fig. 7f). Finally, primary islets were isolated from each group and treated with glucose or KCl in vitro. Results showed that treatment of 16.7 mM glucose induced a 6.5-fold insulin secretion in control islets; this value was decreased to 2.9-fold in *K2fl/fl; MIP-CreERT* islets (Fig. 7g). Activation of β-catenin in β-cells restored the decrease in insulin secretion induced by Kindlin-2 loss. Likewise, treatment of 20 mM KCl stimulated a 7.7-fold insulin secretion in control islets; which was reduced to 3.5-fold in *K2fl/fl; MIP-CreERT* islets. Again, activation of β-catenin in β-cells completely restored the defective insulin secretion in mutant cells. The results revealed that the glucose- and KCl-induced insulin secretion was significantly decreased in *K2*^*f/f*^*; MIP-CreERT* islets compared with that of *MIP-CreERT* islets; this reduction was completely restored in *K2*^*f/f*^*; MIP-CreERT; β-cat (ex3)*^*fl/+*^ islets compared with that of *MIP-CreERT* islets (Fig. 7g). Please note: islets used in these experiments displayed excellent cell viability (Supplementary Fig. 6). Dead cells were scarcely observed in islets of the three genotypes and the morphology of the islets was intact.

## Discussion

The results of our present study establish that Kindlin-2 plays a critical role in regulation of glucose homeostasis during development and homeostasis. In the absence of β-cell Kindlin-2, mice develop high fasting glycemia and fail to properly respond to hyperglycemia. The hyperglycemia seen following Kindlin-2 loss is driven by impairments in GSIS, insulin expression, and β-cell mass, but not by impacting peripheral insulin resistance. Our findings are of broad significance because β-cell failure induced by combination of β-cell loss and impaired insulin secretion from remaining β-cells is a key pathologic basis for diabetes.

In the present study, we demonstrate that Kindlin-2 regulates β-cell function and homeostasis by modulating, at least in part, β-catenin. GSK-3β is a key player in the canonical pathway that regulates the phosphorylation and degradation of β-catenin. Interestingly, we find that Kindlin-2 loss activates GSK-3β by reducing phosphorylation of a negative-regulatory site (Ser9) via undefined mechanism(s), which reduces β-catenin stability and accelerates its degradation in β-cells. The reduced level of β-catenin protein reduces β-cell proliferation and expansion and β-cell mass^13^. This notion is further supported by results from our in vivo rescue experiments in that genetic activation of β-catenin largely restores the defective GTT, GSIS, and β-cell mass induced by the loss of Kindlin-2. Interestingly, Yu et al. reported that Kindlin-2 physically interacted with β-catenin and increased its protein stability to enhance Wnt signaling and promote tumor invasion^42^. Similarly, Qu et al. showed that Kindlin-2 interacted with β-catenin and YB-1 to enhance EGFR transcription during glioma progression^43^. A more recent study by Lin et al. showed that Kindlin-2 promoted hepatocellular carcinoma invasion and metastasis by increasing Wnt/β-catenin signaling^44^. While these in vitro studies suggest a role of the Kindlin-2-β-catenin interaction in regulation of cell functions, in the present study we provide the first in vivo evidence that both factors function in the same genetic pathway in β-cells to regulate glucose homeostasis in mice.

The severe glucose intolerance of the *K2-RIP* animals is in part a consequence of impairment of insulin release from the pancreatic β-cells. Both the first- and second-phase insulin release stimulated by glucose is markedly reduced in the mutant animals. Multiple mechanisms could be involved. Our in vitro study using physiologically relevant primary islets reveals that Kindlin-2 loss impairs the Ca^2+^ influx in β-cells, a critical step for the exocytosis of insulin secretory vesicles. It is likely Kindlin-2 regulates the insulin secretion process through its ability to modulate cytoskeletal remodeling and integrin-ECM interactions, which are all actively involved in modulating insulin granule trafficking and secretion^45–50^. Furthermore, Kindlin-2 signaling maintains a normal β-cell mass via its pro-proliferative and/or pro-survival effects on β-cells. Reduced β-cell mass additionally reduces both the insulin production and secretion in the *K2-RIP* mice. β-Cell deficiency plays an important role in the pathogenesis of both type 1 and type 2 DM. Therefore, restoration or replacement of β-cell function is a long-term goal for the treatment. The Kindlin-2 signaling pathway in β-cells provides a potential therapeutic target for achieving this goal.

In the present study, we provide intriguing and convincing evidence that Kindlin-2 activates insulin expression by interacting with and modulating MafA, a master regulator of insulin gene transcription. First, that loss of Kindlin-2 drastically reduces the level of MafA protein without affecting its mRNA in β-cells suggests a posttranscriptional mechanism. Second, Kindlin-2 activation of the insulin gene promoter is highly dependent on the presence of MafA. Third, Kindlin-2, through its C-terminal region, interacts with MafA and increases its protein stability. The nuclear localization of Kindlin-2 facilitates its interaction with and upregulation of MafA. A putative nuclear localization signal was described in the Kindlin-2 molecule (amino acids 55–72)^51^. Furthermore, Kindlin-2 protein is present in nuclei of the ADTC5 chondrocytes^34^ and the INS-1 pancreatic β cells as demonstrated in this study. Further studies are necessary to define the molecular mechanism(s) responsible for Kindlin-2 nuclear localization. It is known that MafA is phosphorylated at multiple sites by GSK-3β. The phosphorylation of Ser65 in MafA is necessary for the sequential action of GSK-3β on the other sites, acting as the first step for degradation of MafA protein under low-glucose conditions^52–54^. Results from the present study show that Kindlin-2 loss activates GSK-3β by reducing its phosphorylation at Ser9. Thus, it is likely that Kindlin-2 additionally stabilizes MafA protein by inhibiting GSK-3-mediated degradation.

We observed a developmental effect in the mutant islets. The percentage of the β-cells in islets was significantly decreased, while that of the α-cells was concomitantly increased, in mutant islets compared with their control islets. Reduced β-cell proliferation could contribute to this defect.

It is possible that the glucotoxicity, which was initially induced by Kindlin-2 deficiency in β-cells, subsequently exacerbates the diabetic phenotypes in the mutant mice. It should be noted that the level of the fasting blood glucose in *K2-RIP* mice is much lower than that of the *db/db* mice (142 ± 25 mg/dl for *K2-RIP* vs 327 ± 104 mg/dl for *db/db*) (Fig. 2a, Supplementary Fig. 2). Thus, the glucotoxicity should be much subtle in *K2-RIP* mice relative to that in *db/db* mice.

The *RIP-Cre* was reported to target Cre expression in certain areas in the brain^55^. This raises a possibility that Kindlin-2 loss in the brain could contribute to the phenotypes of *K2-RIP* mice. We observed an extremely low level of Kindlin-2 protein in the brain (Supplementary Fig. 7a, b). Furthermore, the levels of Kindlin-2 mRNA and protein were not markedly reduced in *K2-RIP* brain relative to those in control brain (Supplementary Fig. 7c–e). These results suggest that *RIP-Cre* may not target the Kindlin-2-expressing brain cells. It is important to note that, while the *MIP-CreERT* was reported to be not expressed in the brain as demonstrated by the same study^55^, the *K2*^*fl/fl*^*; MIP-CreERT* mice (the present study) did display marked diabetes-like phenotypes (Fig. 6). Collectively, these observations do not support the idea that the diabetic phenotypes displayed by *K2-RIP* mice are due to Kindlin-2 loss in the brain.

Based on findings from this and other studies, we have proposed a working model to explain how Kindlin-2 modulates β cell function and β cell mass (Fig. 7i). Kindlin-2 interacts with and stabilizes key transcription factor MafA in β cells, thus increasing insulin expression. MafA also promotes β cell proliferation and increases β cell mass^56^. In the meantime, Kindlin-2 upregulates the level of β-catenin protein by suppressing GSK-3β activity and/or through a direct interaction with β-catenin^42,43^; this favors β cell proliferation and survival and maintains β cell mass and glucose homeostasis.

It is well established that Kindlin-2 functions as a coactivator of integrins, especially the β integrins, and regulates cell-ECM adhesion, migration and signaling. However, it is interesting to compare the phenotypes from the deletion of Kindlin-2 in β-cells (*K2-RIP*) with those from the deletion of β1 integrin (β*1-RIP*) in the same mouse cell type. First, the defects displayed by *K2-RIP* mice are much more severe than those of the *β1-RIP* mice. *K2-RIP* mice display a severe growth retardation starting at 1 month of age, which is not observed in *β1-RIP* mice^57^. Second, although both genotypes display a reduction in β-cell mass, *K2-RIP* mice exhibit a severe diabetic phenotype, including deficiencies in GTT and GSIS, while *β1-RIP* mice are not diabetic. Results from this study reveal that Kindlin-2 modulates MafA protein and activates *Ins1* gene expression independent of its ability to activate β integrin. Collectively, these findings suggest that Kindlin-2 controls certain function of β-cells independent of β1 integrin activation. Future study will dissect the integrin dependent and independent functions of Kindlin-2 in β-cells.

## Methods

### Reagents

Tissue culture media RPMI 1640 and fetal bovine serum (FBS) were obtained from Thermo Scientific HyClone (Logan, UT, USA). Collagenase Type V, Ficoll PM 400, and glucose were purchased from Sigma-Aldrich (St. Louis, MO, USA), human regular insulin was from Eli Lilly (Indianapolis, IN, USA), and the insulin ELISA Kit from Alpco (Salem, NH, USA) (#80-INSMSU-E01, E10)

### Animal studies

The generation of *Kindlin-2*^*fl/fl*^ mice was recently described^34^. *RIP-Cre* transgenic mice, in which a 668 bp fragment of the rat insulin II gene promoter (*RIP*) drives Cre recombinase expression in β-cells, were purchased from Jackson laboratory (Bar Harbor, ME, USA)^36^. To delete Kindlin-2 expression in β-cells we bred *Kindlin-2*^*fl/fl*^ mice to *RIP-Cre* mice and generated β-cell-selective Kindlin-2 knockout mice (*Kindlin-2*^*RIP*^ or *K2-RIP* mice). *RIP-Cre* littermates were used as controls for this study. To delete Kindlin-2 expression in adult β-cells, we bred *Kindlin-2*^*fl/fl*^ mice with *MIP-Cre/ERT* transgenic mice and generated inducible conditional Kindlin-2 knockout mice (*K2*^*f/f*^*; MIPCreERT*). TM (Sigma T5648) was administrated to 3-month-old mice through a daily peritoneal injection at the dosage of 100 mg/kg body weight for 5 days. This TM regimen dramatically reduced Kindlin-2 protein expression in islet cells (Fig. 6a). To activate β-catenin signaling in β-cells, we bred *K2*^*f/f*^*; MIPCreERT* with *β*-catenin (*ex3*)^*fl/fl*^ mice and generated *K2*^*f/f*^*; MIPCreERT*; *β-cat (ex3)*^*fl/+*^ mice, in which one copy of β-catenin exon 3 gene was floxed. *β-catenin (ex3)*^*fl/fl*^ mice that harbor a mutant β-catenin allele whose exon 3 was floxed by loxP sequences were previously described ^58^. Both *β-catenin (ex3)*^*fl/fl*^ and *β-catenin (ex3)*^*fl/+*^ mice were fertile and normal in body size. All research protocols were approved by the respective Institutional Animal Care and Use Committees of Rush University or Southern University of Science and Technology. We affirm that we have complied with all relevant ethical regulations for animal testing and research in this study.

### Lentiviral shRNA knockdown in primary human pancreatic islets

Human pancreata were obtained from organ procurement organizations following formal research consent and then transported to the UVA Islet Microfluidic Laboratory of the University of Virginia for islet isolation. Human pancreatic islets were isolated by the UVA Islet Microfluidic Laboratory of the University of Virginia and used in this study. Human pancreatic islets were infected with Kindlin-2 shRNA (Sh-K2) or control (Sh-con) lentiviruses^41^. Two days after infection, protein extracts were isolated from both groups and subjected to Western blot analyses with indicated antibodies. The study protocol regarding the work with human islets in this study was approved by the Institutional Review Board of the University of Virginia.

### GTT and GSIS and ITT assays

GTTs were performed on overnight-fasted (14–16 h) mice by i.p. injection of glucose (2 g/kg of body weight). Blood glucose levels were measured at 0, 30, 60, 90, and 120 min after glucose injection using a glucose meter (Bayer HealthCare LLC, Mishawaka, IN, USA). For GSIS, mice were fasted overnight and injected intraperitoneally with glucose (2 g/kg body weight). Blood insulin levels were measured using an ELISA kit from Alpco (Salem, NH, USA) at the indicated time points after glucose injection. ITTs were performed on fasted mice by the i.p. injection of recombinant human insulin (Indianapolis, IN, USA) at a dose of 1 U/kg of body weight; blood glucose levels were measured at 0, 15, 30, 45, and 60 min after insulin injection.

### Isolation of pancreatic islets

Pancreatic islets isolation was performed as previously described^59^. Briefly, mice were sacrificed immediately before harvest. A laparotomy was performed, and the liver reflected back against the diaphragm. The common bile duct was located and clamped at the papilla of Vater. A 27G needle was inserted into the bile duct, and 2–3 ml cold collagenase type V (Sigma, #C9263) solution [1 mg/ml in Hanks buffered salt solution (HBSS)] was injected until the pancreas was visibly distended, followed by digestion for 20 min at 37 °C. The pancreas was then removed by gently tearing/cutting away from the attachment points. The digested pancreas tissues were washed three times using HBSS containing 0.2% bovine serum albumin (BSA), and subjected to discontinuous Ficoll gradient centrifugation. Islets were picked up manually under a dissecting microscope (Nikon Instruments Inc, Melville, NY, USA).

### β-cell area and β-cell mass

Pancreatic sections were subjected to immunohistochemical staining for insulin as previously described^60^. Briefly, pancreases were dissected, weighed, and fixed in 4% paraformaldehyde overnight at 4 °C. Tissues were then transferred to 70% ethanol and subsequently processed for paraffin sections. Five-micrometer sections were dewaxed and hydrated, and antigen retrieval was performed using 10 mM sodium citrate buffer (pH 6.0) at 95 °C for 10 min. Sections were incubated with anti-insulin (1:500; Abcam, ab7842) antibodies overnight at 4 °C in a humidified atmosphere. After washing, rabbit anti-guinea pig IgG/HRP (1:200; Dako, P0141) was added and incubated at room temperature for 45 min. Signals were developed using the EnVision+System-HRP (DAB) kit (Dako North America Inc.) according to the manufacturer’s instruction. Sections were then counterstained with hematoxylin. Pancreatic sections were imaged using a light microscopy (Leica Microsystems, Inc.). The insulin-positive area and pancreatic area were measured using Image Pro Plus 7.0 software (Rockville, MD, USA). The islet β-cell mass was examined using the following equation as described previously^61^: β-cell mass was calculated by dividing total insulin-positive area by total pancreatic area, multiplied by pancreas tissue weight. Approximately 6–8 sections obtained from each tissue were analyzed.

### Cell proliferation and apoptosis

Pancreatic sections from each group of mice were stained with antibodies against insulin or Ki67. Briefly, pancreases were dissected and fixed in 4% paraformaldehyde overnight at 4 °C. Tissues were then transferred to 70% ethanol and processed for paraffin sections. Five-micrometer sections were dewaxed and hydrated. For Ki67 and insulin staining, antigen retrieval was performed using 10 mM sodium citrate buffer (pH 6.0) at 95 °C for 15 min. Slides were incubated with anti-insulin (1: 200; Abcam, ab7842) and anti-Ki67 (1:100; Abcam, ab15580) antibodies overnight at 4 °C in a humidified atmosphere. After washing, appropriate Alexa Fluor 488-labeled goat anti-guinea pig IgG (1:200; Invitrogen, A11073) and Alexa Fluor 594-labeled donkey anti-rabbit IgG (1:2000; Invitrogen, A-21207) secondary antibodies were added and incubated at room temperature for 1 h, followed by counterstaining with DAPI for nuclei. The slides were washed and mounted using a SlowFade^TM^ Antifade kit (Invitrogen, #S2828). The sections were examined using an LSM 700 laser scanning fluorescence confocal microscope running ZEN software (Zeiss). For quantitative analysis, ImageJ (NIH) was used to count the number of DAPI positive cells within the insulin staining area. Insulin-positive cells and Ki67-positive cells were determined. Ki67-positive cells were normalized to total insulin-positive cells in the same area. Cell survival was evaluated using the In Situ Cell Death Detection Kit (Roche Applied Sciences, 11684795910) following the instructions from the manufacturer^34^. This method is based on the classical terminal deoxynucleotidyl transferase dUTP nick end labeling (Tunel) assay to examine apoptosis by detecting DNA fragmentation. Briefly, after dewaxation, antigen retrieval was performed using proteinase K (10 μg/ml in 10 mM Tris/HCl, pH 7.4) at room temperature for 15 min. The slides were washed twice with 1× PBS and incubated in TUNEL reaction mixture (50 μl of Enzyme solution plus 450 μl Label Solution) for 60 min at 37 °C. The pancreas sections were further stained with an antibody insulin (1:200; Abcam, ab7842) and DAPI. The sections were examined using an LSM 700 laser scanning fluorescence confocal microscope running ZEN software (Zeiss). TUNEL-positive cells were normalized to total insulin-positive cells in the same area.

### Measurement of Ca^2+^ influx in primary islets

Islets attached to fibronectin (10 μg/ml, Sigma, #F4759)-coated cover slips were cultured for 1 day and then loaded with the Ca^2+^-sensitive dye Fluo-4 AM (Invitrogen, Carlsbad, CA, USA, #F14201, 10 μM) and 0.1% Pluro (Invitrogen, Carlsbad, CA, USA, P3000MP) in image solution (containing 140 mM NaCl, 5 mM KCl, 5 mM NaHCO_3_, 1 mM MgCl_2_, 10 mM HEPES pH 7.4, and 2 mM CaCl_2_) for 40 min at 37 °C. The cover slips with attached islets were placed to a perfusion chamber with a perfusion rate of 1.5 ml/min. Ca^2+^ was measured using confocal microscopy (Nikon A1R, Nikon Corporation, Melville, NY, USA). Fluo-4 emission signals (F) were background subtracted and normalized to baseline fluorescence (F0), and changes of [Ca^2+^]i are presented as F/F0^62^.

### High-fat diet treatment

Six-week-old male C57BI/6J mice were fed with standard chow or high-fat diet chow for 3 months. Both standard chow (ssniff R/MH) and high-fat diet chow (ssniff EF acc. D12492 (I) mod.) were obtained from sniff Spezialitaten GmbH (Soest, Germany).

### GSIS assays in isolated islets

Islets were isolated and preincubated in 24-well plates for 2.5 h in RPMI 1640 media containing 10% FBS and 2.5 mM glucose at 37 °C and 5% CO_2_. After preincubation, islets were incubated in fresh media containing 16.7 mM glucose solutions at 37 °C and 5% CO_2_ for 30 min, the islets were sedimented and the supernatant was collected. The total protein in the islets was extracted by sonication in 300 μl acid/ethanol (0.18 M HCl in 95% ethanol). The levels of insulin in supernatant and cell extracts were determined using an insulin ELISA kit (ALPCO, Cat#80-INSMSU-E01) according to the manufacturer’s instruction^63^.

### DNA constructs and transfection and infection

pCMV/Flag-Kindlin-2 and pGEX/GST-Kindlin-2 fusion protein expression vectors were described previously^64^. To generate pCMV/Flag-Kindlin-2 expression plasmids expressing truncated forms of Kindlin-2, DNA elements encoding respective Kindlin-2 regions (aa 1–239, aa 1–569, aa 570–680) obtained by PCR were subcloned into the KpnI/XhoI sites of pcDNA3.1(+)-3FLAG vector. The full-length GST-MafA fusion protein expression plasmid was constructed by subcloning the full-length MafA cDNA into the GST gene fusion vector pGEX-4T1 (Amersham Biosciences, Little Chalfont Buckinghamshire, UK) in correct reading frame. The pCMV/β-gal expression vector has been described^65^. The HA-MafA expression vector was constructed by subcloning a full-length mouse MafA cDNA, which was obtained by PCR using a pCMV/MafA plasmid as a template, into the HindIII/Xba1 sites of pcDNA3.1(+)-3HA vector in the correct reading frame. The pCMV/MafA template plasmid was kindly provided by Dr Takaaki Matsuoka of the Osaka University School of Medicine^66^. The rat 460-bp *Ins1-luc* reporter plasmid (*p460rIns1-luc*) was kindly provided by Dr Michael S. Lan of the Louisiana State University Health Sciences Center^39^. The *p460rIns1C1mt-luc*, which contains 4-bp substitution mutations (from TTACAGCTTCAGCC to TTACAGCTTactaC) in the MafA-binding site (C1) at positions -118/-122^38,67^, was generated from *p460rIns1-luc* by PCR amplification using a QuickChange^TM^ XL Site-Directed Mutagenesis Kit (Stratagene, La Jolla, CA, USA) according to the manufacturer’s instructions. All sequences were verified by automatic DNA sequencing.

For transfection, cells were plated on 35-mm dishes at a density of 5 × 10^4^ cells/cm^2^. After 24 h, cells were transfected with Lipofectamine 2000 (Invitrogen, Carlsbad, CA, USA) according to the manufacturer’s instructions. Each transfection contained 0.25 μg of the indicated reporter plasmids plus 1 ng of pRL-SV40, containing a cDNA for Renilla reformis luciferase to control for transfection efficiency. The cells were harvested and assayed using the Dual-Luciferase Assay Kit (Promega, Madison, WI, USA) on a GloMax multi detection system (Promega, Madison, WI, USA). For all transfection experiments, the amount of plasmid DNA was balanced as necessary with pCMV/β-gal such that the total DNA was constant for each group.

Adenoviral vectors for enhanced green fluorescent protein (Ad/EGFP) and Kindlin-2 (Ad/Kindlin-2) were previously described^68^. Ad/Cre was previously described^34^. Cells were infected with adenovirus as previously described^69^. Briefly, Ad/EGFP or Ad/Kindlin-2 was added to INS-1 cells in 1% FBS and incubated for 1 h at 37 °C. Dishes were rotated every 5 min for the first 15 min to ensure that all of the cells were exposed to virus. After 1 h, media were aspirated, and cultures were rinsed twice with serum-free medium, and then fresh media supplemented with 10% FBS were added to the dishes. The amount of adenovirus was balanced as necessary with Ad/EGFP such that the total amount was constant in each group.

### Quantitative real-time RT-PCR and Western blot analyses

RNA was isolated using the RNeasy Mini Kit (QIAGEN, Germantown, MD, USA, Cat#74104) according to the manufacturer’s instruction. Reverse transcription (RT) was performed using 1 μg of denatured RNA and 100 pmol of random hexamers (Applied Biosystem, Foster, CA, USA) in a total volume of 25 μl containing 12.5 U MultiScribe reverse transcriptase (Applied Biosystem, Foster, CA, USA). Quantitative real-time RT-PCR (qPCR) analysis was performed to measure the relative mRNA levels using the SYBR Green kit (Bio-Rad Laboratories Inc, Germantown, MD, USA). Samples were normalized to *Gapdh* expression. The DNA sequences of primers used for qPCR are summarized in Supplementary Tables 1 and 2. Western blot analysis was performed as previously described^70^. Briefly, protein extracts were fractionated on a 10% SDS-PAGE gel and transferred onto nitrocellulose membranes (Schleicher & Schuell, Keene, NH, USA). The membrane was blocked in 5% nonfat milk in Tris-buffered saline/Tween 20 buffer; probed with primary antibodies, followed by incubation with secondary antibodies conjugated with horseradish peroxidase; and visualized using a Western Blotting Detection Kit (GE Healthcare, Chicago, IL, USA, cat#: RPN2106). Antibodies used in this study are listed in Supplementary Table 3.

### IHC and IF staining and confocal analysis

For IHC, 5-μm sections were stained with antibodies or control IgG using the EnVision+System-HRP (DAB) kit (Dako North America Inc, Carpinteria, CA, USA) according to the manufacturer’s instruction. For IF, cells were seeded and cultured on sterile glass cover slips in six-well plates. After 24 h, cells were fixed in 10% formaldehyde for 30 min at 37 °C. Cells were permeabilized with 0.2% Triton X-100 containing DAPI for 5 min, and blocked with 2% BSA for 1 h. The cells were then stained with a primary antibody (Supplementary Table 3) overnight at 4 °C. After washing, cells were incubated with anti-mouse Alexa Fluor 594 (Invitrogen, Carlsbad, CA, USA) secondary antibodies (1:300) for 1 h at room temperature. Cells were then imaged using a confocal microscope (SP2-AOBS Leica Microsystems, Wetzlar, Germany).

### Statistical analysis

The sample size for each experiment was determined based on our previous experience. Animals used in experiments of this study were randomly grouped. IHC, IF and histology were performed and analyzed in a double blinding way. Unpaired Student’s *t* test (two groups) and two-way ANOVA (multiple groups) were used. Results are expressed as mean ± standard deviation (s.d.), as indicated in the figure legends. Differences with *P* < 0.05 were considered statistically significant.

### Reporting summary

Further information on research design is available in the Nature Research Reporting Summary linked to this article.

## Supplementary information

Supplementary Information

Reporting Summary

## Data Availability

All data generated for this study are available from the corresponding authors upon reasonable request. The source data underlying Figs. 1d–f, i, 2a–i, 3a, b, d–g, i, j, 4b–d, 5b, d–k, m–p, 6c–i, and 7b–g and Supplementary Figs. 1a–f, 2, 3a–c, 4a–d, 5, and 7a–e are provided as a Source Data file.

## Source data

Source Data
