# Supplementary material for: Kindlin-2 modulates MafA and β-catenin expression to regulate β-cell function and mass in mice
Source: Nat Commun. 2020 Jan 24;11:484. doi: 10.1038/s41467-019-14186-y (PMC6981167; doi:10.1038/s41467-019-14186-y)
Supplement: Supplementary file 1 — Supplementary Information [file 41467_2019_14186_MOESM1_ESM.pdf]

Supplementary Information

Kindlin-2 modulates MafA and β-catenin expression to regulate β-cell function and mass in mice (NCOMMS-18-01626E)

Zhu et al

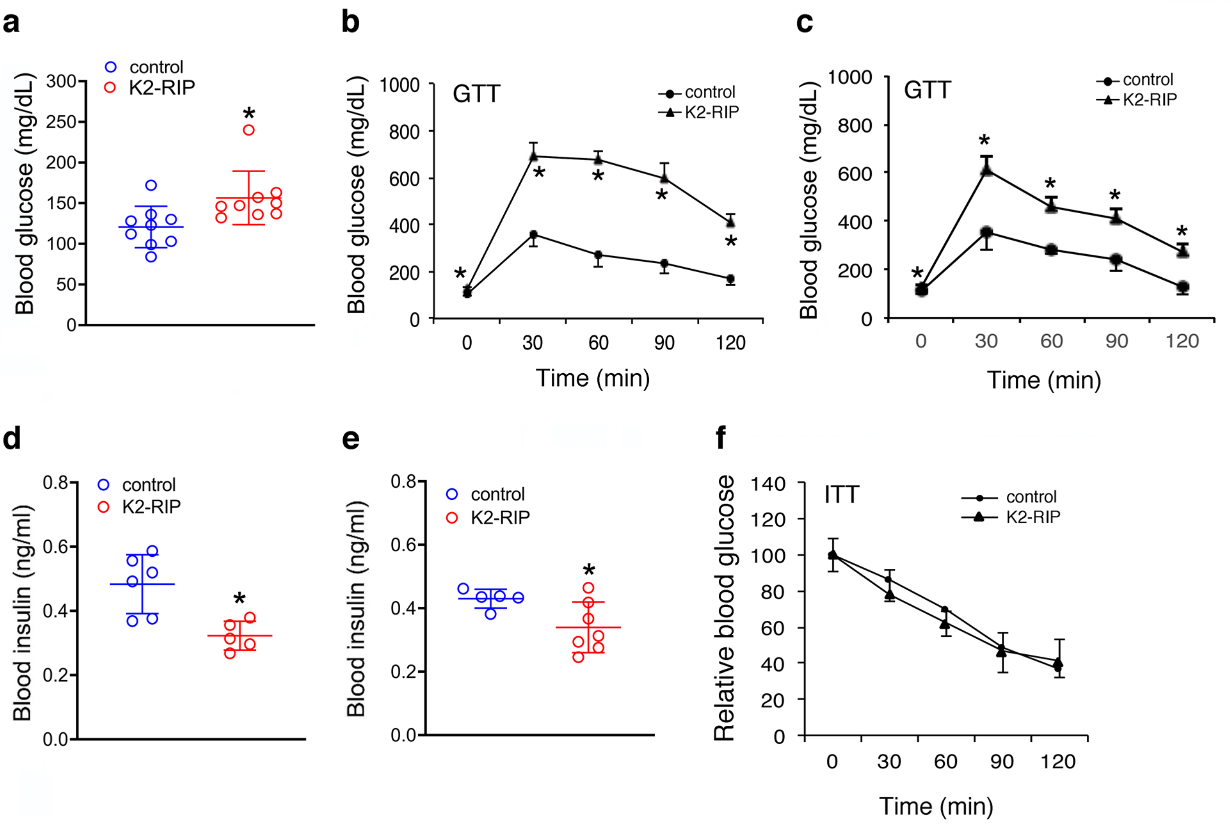

Supplementary Figure 1. Deletion of β-cell Kindlin-2 leads to glucose intolerance.
(a) Fasting blood glucose level. Four-mo-old RIP-Cre (control) and K2-RIP male mice were fasted overnight and tail blood glucose levels were measured. *P < 0.05, N = 9 mice per genotype, Student's t-test. Results are expressed as mean ± standard deviation (s.d.). (b) Glucose tolerance test (GTT). Four-mo-old control and K2-RIP male mice were fasted overnight, followed by performance of GTT assays. *P < 0.05, N = 10 mice per genotype, Student's t-test. Results are expressed as mean ± s.d. (c) Two-mo-old control and K2-RIP female mice were subjected to GTT as in (b). *P < 0.05, N = 8 mice per group, Student's t-test. Results are expressed as mean ± s.d. (d) Fasting blood insulin level. Two-mo-old control and K2-RIP female mice were fasted 6-7 h and tail blood insulin levels were measured by enzyme-linked immunosorbent assay (ELISA). *P < 0.05, versus control, N = 6 for control, N = 5 mice K2-RIP, Student's t-test. Results are expressed as mean ± s.d. (e) Four-mo-old control and K2-RIP male mice were fasted 6-7h. Blood insulin levels were measured by ELISA. * P < 0.05, N = 5 for control, N = 7 mice K2-RIP, Student's t-test. Results are expressed as mean ± s.d. (f) Insulin tolerance test (ITT). Four-mo-old control and K2-RIP male mice were fasted overnight, followed by performance of ITT assays. N = 6 mice per genotype. Results are expressed as mean ± s.d. Source data for Supplementary Figure 1a-f are provided as a Source Data file.

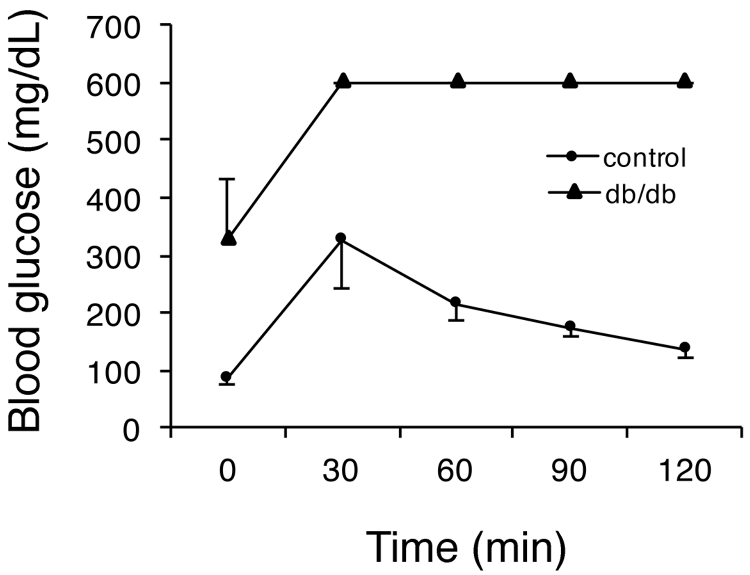

Supplementary Figure 2. Increased fasting glucose level and abnormal GTT in db/db mice. Three-mo-old control and db/db male mice were fasted overnight and given a single intraperitoneal (i.p.) injection of glucose (2 g/kg body weight), followed by measurement of blood glucose levels at 0, 30, 60, 90, and 120 min after glucose injection. *P < 0.05, N = 10 mice for both control and db/db mice, Student's t-test. Results are expressed as mean ± s.d. Please note: when we plotted this figure, we set the blood glucose levels of db/db mice at 30, 60, 90, and 120 min as 600 mg/dl since the blood glucose levels of db/db mice at those time points were so high that exceeded the meter limit (i.e., 600 mg/dl). Source data for Supplementary Figure 2 are provided as a Source Data file.

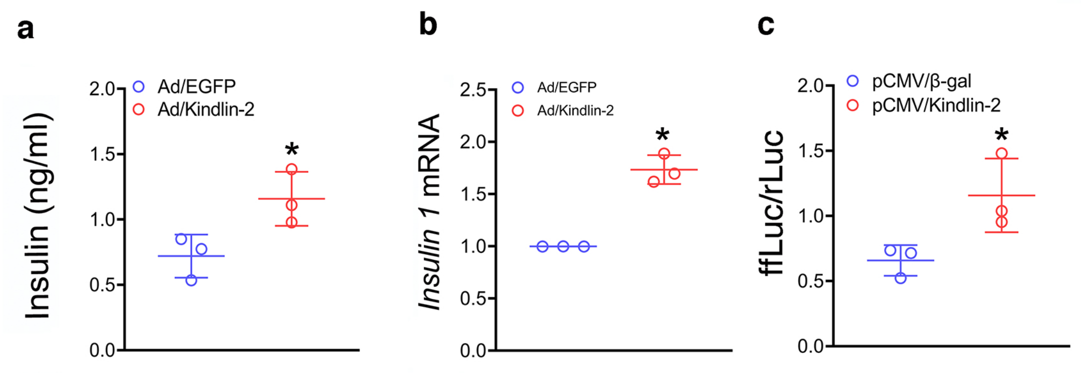

Supplementary Figure 3. Overexpression of Kindlin-2 activates insulin expression in INS-1 cells in vitro. (a,b) INS-1 cells were infected with equal amounts of adenoviral vectors for enhanced green fluorescent protein (Ad/EGFP) or Ad/Kindlin-2, followed by measurement of insulin protein levels in the culture media by ELISA (a) or qPCR analyses (b) for Insulin mRNA expression, which was normalized to Gapdh mRNA level. *$P < 0.05$, versus Ad/EGFP, Results are expressed as mean ± standard deviation (s.d.). Statistical analyses (Student's t-test) were performed using the average values of triplicates from three independent experiments. (c) INS-1 cells were co-transfected with p460rINS1-luc, pRL-SV40 (for normalization), and the indicated amounts of pCMV/Kindlin-2 or pCMV/β-gal, followed by dual-luciferase assays. The amount of plasmid DNA was balanced as necessary with pCMV/β-gal such that the total DNA was constant for each group. *$P < 0.05$, versus pCMV/β-gal. Results are expressed as mean ± s.d. Statistical analyses (Student's t-test) were performed using the average values of triplicates from three independent experiments. Source data for Supplementary Figure 3a-c are provided as a Source Data file.

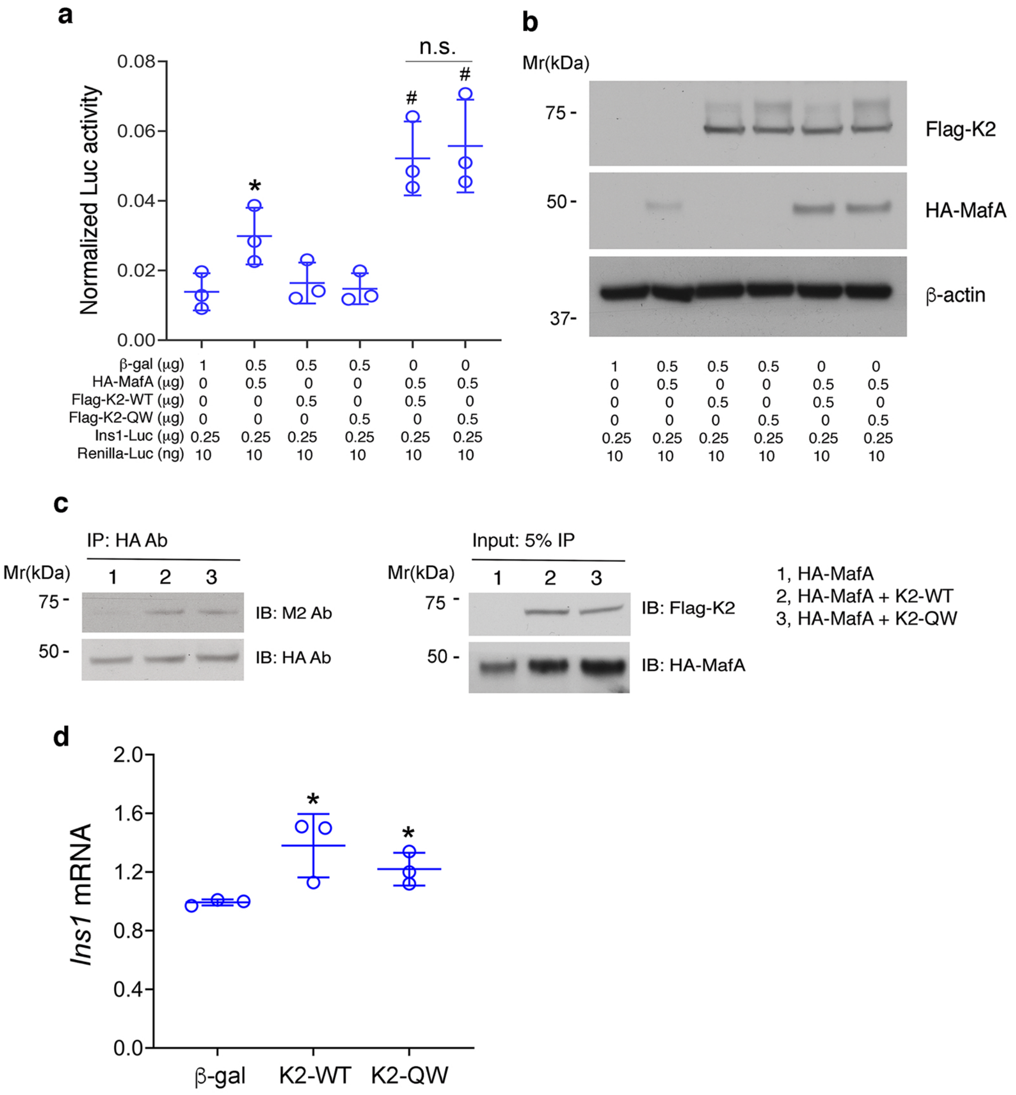

Supplementary Figure 4. Kindlin-2 interacts with and upregulates MafA and activates insulin expression independent of integrin activation. (a,b) COS-7 cells were co-transfected with p460rINS1-luc, pRL-SV40 (for normalization), and the indicated amounts of pCMV/HA-MafA, wild-type pCMV/Flag-Kindlin-2 (K2-WT) and an integrin-binding defective pCMV/Flag-Kindlin-2 (K2-QW), followed by dual-luciferase assays (a) or western blotting (b). The amount of plasmid DNA was balanced as necessary with pCMV/β-gal such that the total DNA was constant for each group. *P < 0.05, versus pCMV/β-gal, #P < 0.05, versus pCMV/HA-MafA, Student's t-test. Results are expressed as mean ± standard deviation (s.d.). Statistical analyses (Student's t-test) were performed using the average values of triplicates from three independent experiments. (c) Immunoprecipitation (IP) assay. Whole cell extracts from COS-7 cells overexpressing HA-MafA and wild-type Flag-Kindlin-2 (K2-WT) or an integrin-binding defective pCMV/Flag-Kindlin-2 (K2-QW) were immunoprecipitated with human influenza hemagglutinin (HA) antibody followed by western blot analysis using M2 (top) or HA (bottom) antibody. (d) INS-1 cells were transfected with equal amounts of pCMV/β-gal, wild-type pCMV/Flag-Kindlin-2 (K2-WT) and an integrin-binding defective pCMV/Flag-Kindlin-2 (K2-QW), followed by qPCR analyses for Insulin mRNA expression, which was normalized to Gapdh mRNA level. *P < 0.05, versus pCMV/β-gal. Results are expressed as mean ± s.d. Statistical analyses (Student's t-test) were performed using the average values of triplicates from three independent experiments. Source data for Supplementary Figure 4a-d are provided as a Source Data file.

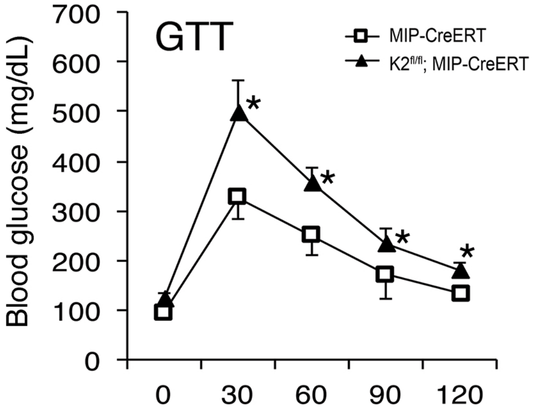

Supplementary Figure 5. Inducible deletion of Kindlin-2 in adult β cells causes glucose intolerance in female mice. Glucose tolerance test (GTT). Three-mo-old K2f/f; MIPCreERT and control female mice were treated with tamoxifen (TM) as described in Methods. After 30 d after injection, mice were fasted overnight, followed by performance of GTT assays. *P < 0.05, versus control, N = 4 mice for control, N = 7 mice for K2f/f; MIPCreERT, Student's t-test. Results are expressed as mean ± standard deviation (s.d.). Source data for Supplementary Figure 5 are provided as a Source Data file.

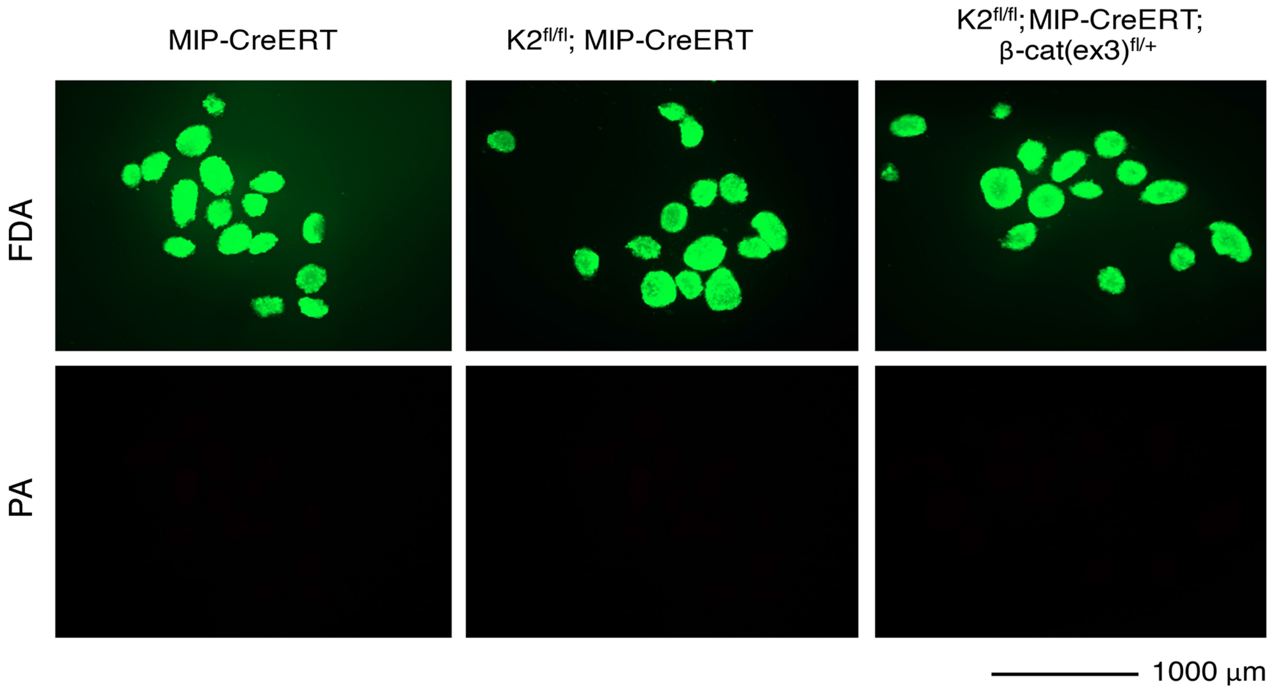

Supplementary Figure 6. Pancreatic islet cell viability. Primary pancreatic islets from MIP-CreERT, K2fl/fl; MIP-CreERT, and K2fl/fl; MIP-CreERT; β–cat(ex3)fl/+ mice were stained with fluorescein diacetate (FDA) for live cells (green) or propidium iodide (PI) for dead cells (red). Source data for Supplementary Figure 6 are provided as a Source Data file.

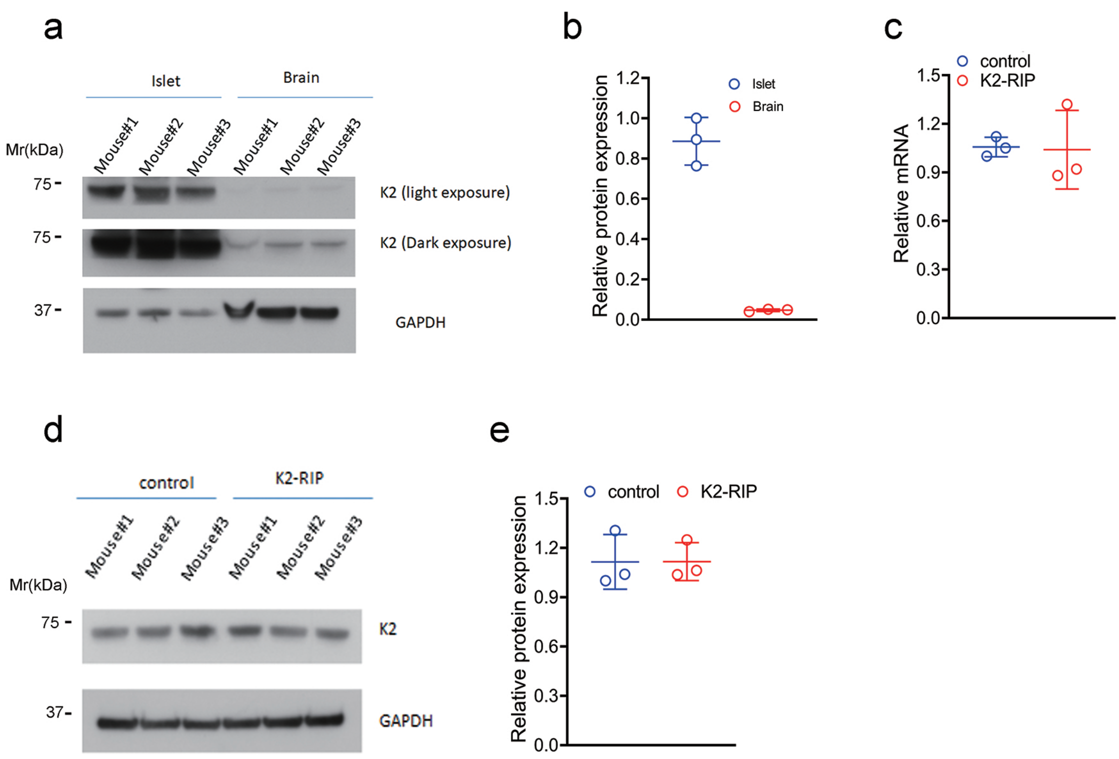

Supplementary Figure 7. Kindlin-2 expression is not reduced in K2-RIP brain. (a) Western blotting. Protein extracts isolated from primary pancreatic islets or the brains of 1-mo-old normal C57BL/6 male mice were subjected to western blotting for Kindlin-2 expression. Gapdh was used as a loading control. Quantitative data (b). Student's t-test was performed. N = 3 mice per group. Results are expressed as mean ± s.d. (c) Quantitative real-time reverse transcriptase-polymerase chain reaction (qPCR) analyses. Total RNAs isolated from 2-mo-old male K2-RIP mice or control (RIP-Cre) brains were subjected to qPCR analyses for Kindlin-2 expression. Kindlin-2 mRNA was normalized to Gapdh mRNA. Student's t-test was performed using the average values of triplicates. N = 3 mice per group. (d,e) Western blotting. Protein extracts isolated from 2-mo-old male K2-RIP mice or control (RIP-Cre) brains were subjected to western blotting for Kindlin-2 expression. Gapdh was used as a loading control. Quantitative data (e). Student's t-test was performed. N = 3 mice per group. Results are expressed as mean ± s.d. Source data for Supplementary Figure 7 are provided as a Source Data file.

Supplementary Table 1: Mouse qPCR primers

Name	5' primer	3' primer
Gapdh	CAGTGCCAGCCTCGTCCCGTAGA	CTGCAAATGGCAGCCCTGGTGAC
Insulin 1	GCAAGCAGGTCATTGTTTCAAC	AAGCCTGGGTGGGTTTGG
Kindlin-2	TGGACGGGATAAGGATGCCA	TGACATCGAGTTTTTCCACCAAC
MafA	GCTTCAGCAAGGAGGAGGTC	TCTCGCTCTCCAGAATGTGC

Supplementary Table 2: Rat qPCR primers

Name	5' primer	3' primer
GAPDH	CCTTCCGTGTTCCTACCCCCAAT	GCCCAGGATGCCCTTTAGTGG
INSULIN 1	ACACCCAAGTCCCGTCGTGAAGTG G	GGCGGGGAGTGGTGGACTCAGT

Supplementary Table 3: Antibodies information

Antibody	Company	Catalog #	Application/Dilution
β-Actin	Sigma	A2228	WB (1:10000)
Active-β-catenin	Millipore	05-665	WB (1:1000)
β-Catenin	CST	9562	WB (1:1000)
Glucagon	CST	2760	IF (1:100)
Gapdh	Santa Cruz	Sc-32233	WB(1:5000)
Glucagon	Sigma	G2654	IF (1:50)
Gsk-3β	Santa Cruz	sc-9166	IHC (1:100), WB (1:1000)
p-Gsk-3β (Ser9)	Santa Cruz	sc-11757	IHC (1:100), WB (1:1000)
Insulin	Abcam	ab7842	IHC (1:600), IF (1:200)
Ki67	Abcam	ab15580	IF (1:100)
Kindlin-2	Abcam	Ab74030	WB (1:1000), IHC (1:2000), IF (1:50)
MafA	Bethyl	IHC-00352	IHC (1:500)
MafA	Bethyl	A300-611A	WB (1:1000)
Pdx1	Abcam	Ab47267	IHC (1:1000)
α-Tubulin	Sigma	T9026	WB (1:5000)
