# Supplementary material for: Kindlin-2 modulates MafA and β-catenin expression to regulate β-cell function and mass in mice
Source: Nat Commun. 2020 Jan 24;11:484. doi: 10.1038/s41467-019-14186-y (PMC6981167; doi:10.1038/s41467-019-14186-y)
Supplement: Supplementary file 2 — Reporting Summary [file 41467_2019_14186_MOESM2_ESM.pdf]

nature research

Corresponding author(s): Guozhi Xiao

Last updated by author(s): Dec 11, 2019

Reporting Summary

Nature Research wishes to improve the reproducibility of the work that we publish. This form provides structure for consistency and transparency in reporting. For further information on Nature Research policies, see Authors & Referees and the Editorial Policy Checklist .

Statistics

For all statistical analyses, confirm that the following items are present in the figure legend, table legend, main text, or Methods section.

n/a	Confirmed	
☐	☒	The exact sample size (n) for each experimental group/condition, given as a discrete number and unit of measurement
☐	☒	A statement on whether measurements were taken from distinct samples or whether the same sample was measured repeatedly
☐	☒	The statistical test(s) used AND whether they are one- or two-sided<br>*Only common tests should be described solely by name; describe more complex techniques in the Methods section.*
☒	☐	A description of all covariates tested
☒	☐	A description of any assumptions or corrections, such as tests of normality and adjustment for multiple comparisons
☐	☒	A full description of the statistical parameters including central tendency (e.g. means) or other basic estimates (e.g. regression coefficient) AND variation (e.g. standard deviation) or associated estimates of uncertainty (e.g. confidence intervals)
☒	☐	For null hypothesis testing, the test statistic (e.g. F, t, r) with confidence intervals, effect sizes, degrees of freedom and P value noted<br>*Give P values as exact values whenever suitable.*
☒	☐	For Bayesian analysis, information on the choice of priors and Markov chain Monte Carlo settings
☒	☐	For hierarchical and complex designs, identification of the appropriate level for tests and full reporting of outcomes
☒	☐	Estimates of effect sizes (e.g. Cohen's d, Pearson's r), indicating how they were calculated

Our web collection on statistics for biologists contains articles on many of the points above.

Software and code

Policy information about availability of computer code

Data collection	n/a
Data analysis	n/a

For manuscripts utilizing custom algorithms or software that are central to the research but not yet described in published literature, software must be made available to editors/reviewers. We strongly encourage code deposition in a community repository (e.g. GitHub). See the Nature Research guidelines for submitting code & software for further information.

Data

Policy information about availability of data

All manuscripts must include a data availability statement. This statement should provide the following information, where applicable:

- Accession codes, unique identifiers, or web links for publicly available datasets
- A list of figures that have associated raw data
- A description of any restrictions on data availability

A data availability statement (All data generated for this study are available from the corresponding authors upon reasonable request. The source data underlying Figs 1d-f, 1i, 2a–i, 3a, 3b, 3d-g, 3i, 3j, 4b-d, 5b, 5d-k, 5m-p, 6c-i, and 7b-g and Supplementary Figs 1a-f, 2, 3a-c, 4a-d, 5, and 7a-e are provided as a Source Data file.") is provided.

Field-specific reporting

Please select the one below that is the best fit for your research. If you are not sure, read the appropriate sections before making your selection.

Life sciences study design

All studies must disclose on these points even when the disclosure is negative.

Sample size	Sample size information (The sample size for each experiment was determined based on our previous experience.) is provided.
Data exclusions	n/a
Replication	Information is provided in legends of each figure.
Randomization	Randomization information (Animals used in experiments of this study were randomly grouped. IHC, IF and histology were performed and analyzed in a double blinding way.) is provided.
Blinding	Blinding information (Animals used in experiments of this study were randomly grouped. IHC, IF and histology were performed and analyzed in a double blinding way.) is provided.

Reporting for specific materials, systems and methods

We require information from authors about some types of materials, experimental systems and methods used in many studies. Here, indicate whether each material, system or method listed is relevant to your study. If you are not sure if a list item applies to your research, read the appropriate section before selecting a response.

Materials & experimental systems

n/a	Involved in the study	
☐	☒	Antibodies
☐	☒	Eukaryotic cell lines
☒	☐	Palaeontology
☐	☒	Animals and other organisms
☒	☐	Human research participants
☒	☐	Clinical data

Methods

n/a	Involved in the study	
☒	☐	ChIP-seq
☒	☐	Flow cytometry
☒	☐	MRI-based neuroimaging

Antibodies

Antibodies used

Required antibodies information is provided in Supplementary Table 3.
Supplementary Table 3: Antibodies information
Antibody Company Catalog # Application/Dilution
b-Actin Sigma A2228 WB (1:10000)
Active-b-catenin Millipore 05-665 WB (1:1000)
β-Catenin CST 9562 WB (1:1000)
Glucagon CST 2760 IF (1:100)
Gapdh Santa Cruz Sc-32233 WB(1:5000)
Glucagon Sigma G2654 IF (1:50)
Gsk-3b Santa Cruz sc-9166 IHC (1:100), WB (1:1000)
p-Gsk-3b (Ser9) Santa Cruz sc-11757 IHC (1:100), WB (1:1000)
Insulin Abcam ab7842 IHC (1:600), IF (1:200)
Ki67 Abcam ab15580 IF (1:100)
Kindlin-2 Abcam Ab74030 WB (1:1000), IHC (1:2000), IF (1:50)
MafA Bethyl IHC-00352 IHC (1:500)
MafA Bethyl A300-611A WB (1:1000)
Pdx1 Abcam Ab47267 IHC (1:1000)
a-Tubulin Sigma T9026 WB (1:5000)

Validation

Information is provided in Supplementary Table 3.
Supplementary Table 3: Antibodies information
Antibody Company Catalog # Application/Dilution
b-Actin Sigma A2228 WB (1:10000)
Active-b-catenin Millipore 05-665 WB (1:1000)
β-Catenin CST 9562 WB (1:1000)
Glucagon CST 2760 IF (1:100)
Gapdh Santa Cruz Sc-32233 WB(1:5000)

Glucagon Sigma G2654 IF (1:50)
Gsk-3b Santa Cruz sc-9166 IHC (1:100), WB (1:1000)
p-Gsk-3b (Ser9) Santa Cruz sc-11757 IHC (1:100), WB (1:1000)
Insulin Abcam ab7842 IHC (1:600), IF (1:200)
Ki67 Abcam ab15580 IF (1:100)
Kindlin-2 Abcam Ab74030 WB (1:1000), IHC (1:2000), IF (1:50)
MafA Bethyl IHC-00352 IHC (1:500)
MafA Bethyl A300-611A WB (1:1000)
Pdx1 Abcam Ab47267 IHC (1:1000)
a-Tubulin Sigma T9026 WB (1:5000)

Eukaryotic cell lines

Policy information about cell lines

Cell line source(s)	Cell line source(s) (We want to thank H. Henry Dong of the University of Pittsburgh for providing the INS-1 cells) is provided.
Authentication	Information (We want to thank H. Henry Dong of the University of Pittsburgh for providing the INS-1 cells) is provided.
Mycoplasma contamination	n/a.
Commonly misidentified lines (See ICLAC register)	n/a

Animals and other organisms

Policy information about studies involving animals; ARRIVE guidelines recommended for reporting animal research

Laboratory animals	Laboratory animals information (The generation of Kindlin-2fl/fl mice was recently described 34. RIP-Cre transgenic mice, in which a 668 bp fragment of the rat insulin II gene promoter (RIP) drives Cre recombinase expression in-cells, were purchased from Jackson laboratory (Bar Harbor, ME, USA). To delete Kindlin-2 expression in beta-cells, we bred Kindlin-2fl/fl mice to RIP-Cre mice and generated beta-cell-selective Kindlin-2 knockout mice (Kindlin-2RIP or K2-RIP mice). RIP-Cre littermates were used as controls for this study. To delete Kindlin-2 expression in adult beta-cells, we bred Kindlin-2fl/fl mice with MIP-Cre/ERT transgenic mice and generated inducible conditional Kindlin-2 knockout mice (K2f/f; MIPCreERT). Tamoxifen (TM, Sigma T5648) was administrated to 3-month-old mice through a daily peritoneal injection at the dosage of 100 mg/kg body weight for 5 days. This TM regimen dramatically reduced Kindlin-2 protein expression in islet cells (Fig. 6a). To activate-catenin signaling in beta-cells, we bred K2f/f; MIPCreERT with beta-catenin (ex3) fl/fl mice and generated K2f/f; MIPCreERT; b-cat (ex3) fl/+ mice, in which one copy of beta-catenin exon 3 gene was floxed. beta-catenin (ex3) fl/fl mice that harbor a mutant beta-catenin allele whose exon 3 was floxed by loxP sequences were previously described 58. Both beta-catenin (ex3) fl/fl and b-catenin (ex3) fl/+ mice were fertile and normal in body size. All research protocols were approved by the respective Institutional Animal Care and Use Committees (IACUC) of Rush University or Southern University of Science and Technology. We affirm that we have complied with all relevant ethical regulations for animal testing and research in this study.) is provided.
Wild animals	n/a
Field-collected samples	n/a
Ethics oversight	Ethics oversight information (All research protocols were approved by the respective Institutional Animal Care and Use Committees (IACUC) of Rush University or Southern University of Science and Technology. We affirm that we have complied with all relevant ethical regulations for animal testing and research in this study.) is provided. The study protocol regarding the work with human islets in this study was approved by the Institutional Review Board (IRB) of the University of Virginia.

Note that full information on the approval of the study protocol must also be provided in the manuscript.
